# Patient-reported outcome measures for life participation in patients with chronic kidney disease: a systematic review

**DOI:** 10.1093/ckj/sfae341

**Published:** 2024-11-12

**Authors:** Anastasia Hughes, Angela Ju, Rosanna Cazzolli, Martin Howell, Fergus J Caskey, Meghan J Elliott, Janine Farragher, Sharlene Greenwood, Adeera Levin, Karine Manera, Amanda Sluiter, Armando Teixeira-Pinto, Hernán Trimarchi, Bill Wang, Chandana Guha, Rebecca Wu, Allison Jauré

**Affiliations:** Sydney School of Public Health, University of Sydney, Sydney, NSW, Australia; Centre for Kidney Research, Children's Hospital at Westmead, Westmead, NSW, Australia; Sydney School of Public Health, University of Sydney, Sydney, NSW, Australia; Centre for Kidney Research, Children's Hospital at Westmead, Westmead, NSW, Australia; Sydney School of Public Health, University of Sydney, Sydney, NSW, Australia; Centre for Kidney Research, Children's Hospital at Westmead, Westmead, NSW, Australia; Sydney School of Public Health, University of Sydney, Sydney, NSW, Australia; Centre for Kidney Research, Children's Hospital at Westmead, Westmead, NSW, Australia; Menzies Centre for Health Policy and Economics, University of Sydney, Sydney, NSW, Australia; Bristol Medical School, University of Bristol, Bristol, UK; University of Calgary, Calgary, AB, Canada; Department of Occupational Science and Occupational Therapy, University of Toronto, Toronto, ON, Canada; King’s College Hospital, NHS Trust, London, UK; King’s College London , London, UK; Division of Nephrology, University of British Columbia, Vancouver, BC, Canada; Sydney School of Public Health, University of Sydney, Sydney, NSW, Australia; Centre for Kidney Research, Children's Hospital at Westmead, Westmead, NSW, Australia; Sydney School of Public Health, University of Sydney, Sydney, NSW, Australia; Centre for Kidney Research, Children's Hospital at Westmead, Westmead, NSW, Australia; Sydney School of Public Health, University of Sydney, Sydney, NSW, Australia; Centre for Kidney Research, Children's Hospital at Westmead, Westmead, NSW, Australia; Nephrology Service and Kidney Transplant Unit, Hospital Británico de Buenos Aires, Buenos Aires, Argentina; International Society of Nephrology, Patient Liaison Advisory Group, Brussels, Belgium; Sydney School of Public Health, University of Sydney, Sydney, NSW, Australia; Centre for Kidney Research, Children's Hospital at Westmead, Westmead, NSW, Australia; Sydney School of Public Health, University of Sydney, Sydney, NSW, Australia; Centre for Kidney Research, Children's Hospital at Westmead, Westmead, NSW, Australia; Sydney School of Public Health, University of Sydney, Sydney, NSW, Australia; Centre for Kidney Research, Children's Hospital at Westmead, Westmead, NSW, Australia

**Keywords:** CKD, kidney disease, life participation, PROMs, PROs

## Abstract

**Background:**

The symptoms, comorbidities and treatment burden associated with chronic kidney disease (CKD) can be debilitating and limit life participation in patients with CKD not requiring kidney replacement therapy (KRT). The aim of this study was to identify the characteristics, content and psychometric properties of patient-reported outcome measures (PROMs) used to assess life participation in patients with CKD.

**Methods:**

We searched MEDLINE, Embase, PsycINFO and CINAHL from database inception to February 2023 for all studies that reported life participation in patients with CKD (stages 1–5 not requiring kidney replacement therapy). We analysed the characteristics, dimensions of life participation and psychometric properties of the measures.

**Results:**

From the 114 studies included, 20 (18%) were randomized trials, 3 (3%) were non-randomized trials and 91 (80%) were observational studies. Forty-one different measures were used to assess life participation, of which six (15%) were author-developed measures. Twelve (29%) measures assessed life participation specifically, while 29 (71%) measures assessed broader constructs such as quality of life, which included questions relevant to life participation. The 36-Item Short Form Health Survey (SF-36) and Kidney Disease Quality of Life Short Form (KDQOL-SF) were the most frequently used, in 39 (34%) and 24 (21%) studies, respectively. Many content domains for life participation were assessed, including physical activities (walking, running and sports), social activities, leisure activities, work or study and self-care. None of the measures for life participation were developed specifically for CKD. Four measures (EuroQol 5-dimension 3-level (EQ-5D-3L), Functional Assessment of Cancer Therapy - Anemia, Short Form 6-dimension and Short-From 36-dimension (SF-36)) had validation data collected in patients with CKD.

**Conclusion:**

The measures for life participation used in patients with CKD vary in content, with few validated in the CKD population. There is a need for a validated measure to assess life participation in a meaningful and consistent way in all patients with CKD worldwide.

## Graphical Abstract

**Graphical Abstract:**
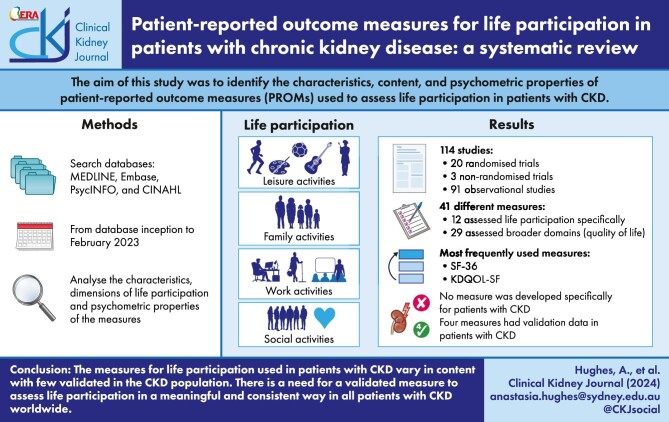

KEY LEARNING POINTS **What was known:**

- Life participation has been identified by patients, caregivers and health professionals as a critically important outcome for patients with CKD. However, there is need for a validated measure to assess life participation.

**This study adds:**

- We identified the characteristics, content and psychometric properties of patient-reported outcome measures used to assess life participation in patients with CKD.
- We found a high degree of uncertainty in the suitability of the measure with further validation required.

**Potential impact:**

- To provide a valid, reliable and robust outcome measure for use in patients with CKD to be reported in all trials.

## INTRODUCTION

Patients with chronic kidney disease (CKD) have an increased risk of mortality, multimorbidities and hospitalization compared with the general population [1–3]. While CKD is often asymptomatic in earlier stages, symptoms including fatigue,

headaches, cramping, pruritus, cognitive impairment and sleep disturbances can become debilitating as the disease progresses [4–6]. These challenges, along with the burden and side effects of treatment can limit life participation in patients with earlier-stage CKD [4–6]. Diagnosis can be a challenging time, as awareness of CKD in the general population is low combined with variable symptoms and prognoses and an uncertain path ahead [4, 7, 8]. This may impact on an individual's ability to fully participate in daily activities, adjust to life with CKD, adhere to treatment and take part in interventions [4, 8]. Patient perspectives and experiences of CKD indicate an inextricable link between mental and physical health [4, 8].

Life participation is defined here as the ability to meaningfully participate in activities of daily living such as work (employment, housework, study), family, social (friends and others) and leisure activities (exercise, hobbies, travel) [9, 10]. These activities provide a sense of fulfilment, enjoyment, autonomy and hope [9]. The concept of life participation focuses on non-self-care activities while remaining broad to allow the patient to subjectively assess their own life participation. Other terms such as ‘social participation’, ‘social function’ and ‘activities of daily living’ have also been used to capture the concept of life participation [10]. Patients with CKD not requiring kidney replacement therapy (KRT) and their caregivers have identified life participation as a critically important outcome [4, 11]. Life participation differs from quality of life, which is defined by the World Health Organization (WHO) ‘as an individual's perception of their position in life in the context of the culture and value systems in which they live and in relation to their goals, expectations, standards and concerns’ [12].

Despite being an outcome of critical importance to patients and caregivers, life participation is rarely included in trials in CKD patients [13], thus the evidence base to support decision-making about interventions to improve life participation in patients with CKD remains lacking. Additionally, the content and psychometric robustness of the measures used in patients with CKD is uncertain. The aim of this systematic review was to identify the characteristics, content and psychometric properties of the patient-reported outcome measures (PROMs) used to assess life participation in the CKD population not requiring KRT, which may guide the identification or development of a psychometrically robust PROM for life participation that is meaningful to CKD patients not requiring KRT.

## MATERIALS AND METHODS

### Selection criteria

We searched for randomized and non-randomized controlled trials and observational studies that included at least one item of a PROM for life participation (ability to meaningfully participate in activities) in CKD patients. Studies were eligible if they included adult patients >18 years of age with CKD not requiring KRT. No time or language restrictions were applied. If studies were not available in the English language they were excluded. Studies that included a measure that assessed broader constructs (e.g. quality of life, health status) were eligible if at least one question focussed on life participation. Studies reporting clinician- or proxy-reported outcomes were excluded. Abstract-only citations were included if they provided adequate information about the measure used to assess life participation.

### Study sources and measures

**Figure 1:**
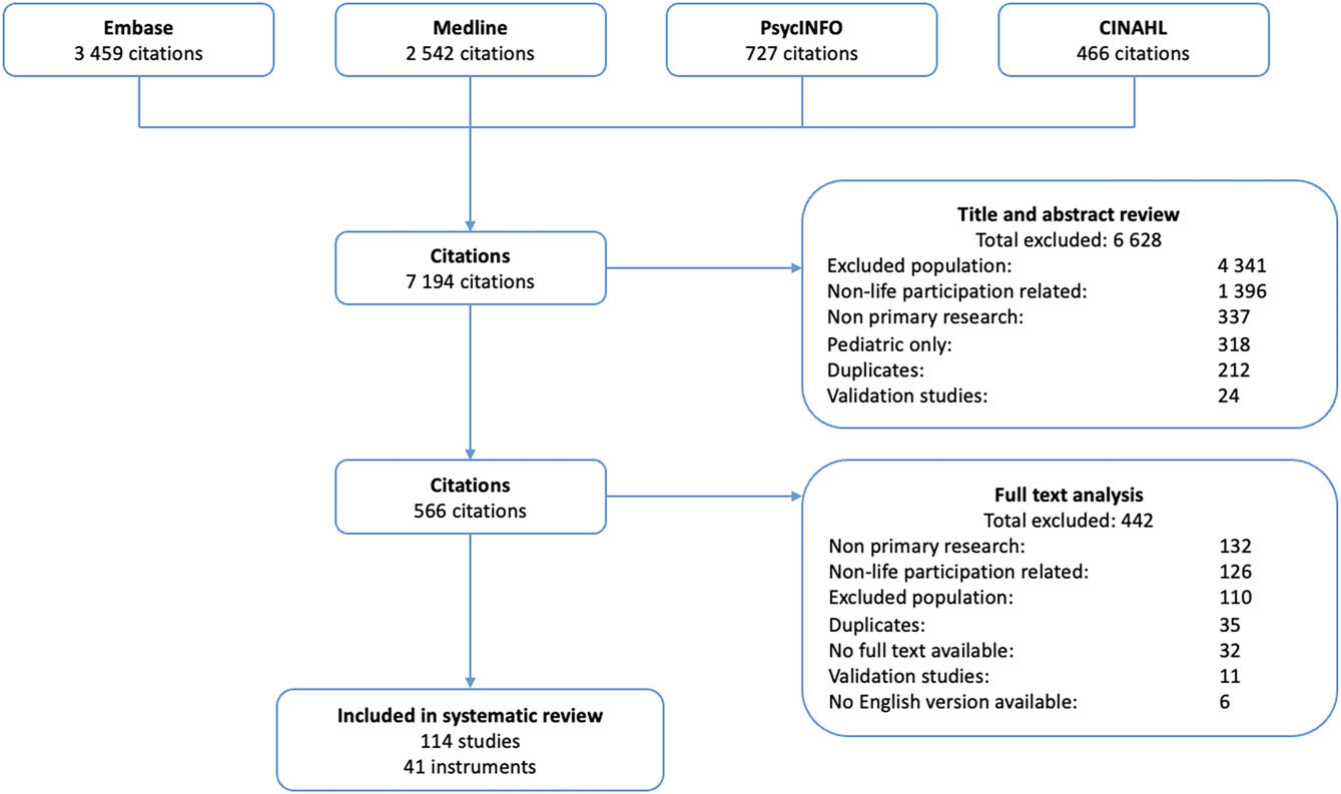
Search results.

We searched the MEDLINE, Embase, PsycINFO and CINAHL databases from their respective database inception to February 2023. The search strategy is provided in Supplementary Table S1. We also searched the reference lists of relevant studies, e.g. systematic reviews of quality of life in CKD. Two authors (A.H. and A.Jaure) screened the results initially based on title and abstract then assessed the remaining by full text. Studies that did not meet the inclusion criteria were excluded (Fig. 1).

### Data extraction and analysis

From each study, A.H. extracted the following information: author, publication, year, sample size (patients with CKD not requiring KRT), country, type of intervention (if applicable) and measure used to assess life participation. For each outcome measure we referred to the source study or program manual to determine the characteristics of each measure, including the response format, number of items, recall period, cost of license to use the measure, completion time and language. A.H. searched for validation work for each measure to extract psychometric data in CKD patients not requiring KRT.

### Content dimensions of life participation

Life participation is comprised of two broad dimensions: obligatory and non-obligatory, which we used to categorize the content of each measure [14, 15]. Obligatory activities include daily activities (eating, washing, self-care), household tasks (cooking, cleaning, lifting/moving items, climbing stairs), employment and study [14, 15]. Non-obligatory tasks include social activities (friends, family, relationships, sex life), sports (running, swimming, walking, bowling, golf), recreational/leisure activities and travel [14, 15]. As life participation is a subjective concept, a patient can assess his/her ability to participate in each of the key domains (work, family, social and leisure) without definitive restrictions to obligatory and non-obligatory dimensions, e.g. cooking may be either obligatory, non-obligatory or both.

### Assessment of psychometric properties

Following the COnsensus-based Standards for the selection of health Measurement INstruments–Core Outcome Measures in Effectiveness Trials framework [16, 17], we analysed the available evidence for validity and reliability (e.g. content validity, criterion validity, cross-cultural validity, known groups validity, structural validity, responsiveness and reliability, including internal consistency and test–retest) of the included measures.

## RESULTS

### Characteristics of the studies

We included 114 studies involving 114 845 patients with CKD across 30 countries. Of the included studies, 20 (18%) were randomized trials, 3 (3%) were non-randomized trials and 91 (80%) were observational studies. The search results are depicted in Fig. 1 and the characteristics of the studies are shown in Supplementary Table S2.

### Characteristics of measures

Across the 114 studies, 41 measures were used to assess life participation. Of these, 27 (66%) measures were used in only one study. The 36-item Short Form (SF-36) was the most frequently used instrument [39 studies (34%)], followed by the Kidney Disease Quality of Life Short Form (KDQOL-SF) [24 studies (21%)] and the World Health Organization Quality of Life brief version (WHOQOL-BREF) [8 studies (7%)]. A detailed summary of characteristics of the measures and frequency of use are provided in Table 1.

**Table 1:** Characteristics of measures used to assess life participation in CKD.

Measure	Response format	Items, *n*	Recall	Cost	Completion time^a^ (minutes)	Specific to LP	Specific to CKD^b^	Language^c^	Frequency of use (number of studies)
15D [36]	5-point ordinal scale	15	Current	No charge	≈5	No	No	Multiple languages including English, Arabic, Chinese, Danish, Dutch, English, French, German and Portuguese	1
Modified BAI [37, 38]	2-/3-/4-point ordinal scale^c^	10	Past 24–48 hours, 7 days	Not stated	2–5	Yes	No	English	1
Chula ADL [39]	2-/3-/4-point Likert	5	Not stated	Not stated	≈1	Yes	No	English	1
DASI [40]	Yes/no	12	Current	Contact author	<3	Yes	No	English	1
EQ-5D [41, 42]	‘Indicate which statement best describes…’, VAS	16	Current	Licensing fee based on quote	<5	No	No	130 languages including English and Chinese	1
EQ-5D-3L [41]	3-point ordinal scale	5	Current	Free for non-commercial use	<5	No	No	>180 languages	7
EQ-5D-5L [42]	5-pont ordinal scale	5	Current	Free for non-commercial use	<5	No	No	>180 languages	4
FACT-An [43]	5-point Likert scale	47	Past 7 days	Non-commercial use assessed per case basis. Licencing fee not typically applied to investigator-led, students and clinical use	10–15	No	No	Multiple languages including, English, Danish, Spanish, French and Chinese. Available translations of the FACT-An can be obtained by registering for permission	1
FACIT-SP [44]	5-point Likert scale	39	Past 7 days	Non-commercial use assessed per case basis. Licencing fee not typically applied to investigator-led, students and clinical use	10–15	No	No	Multiple languages in Dutch, French, English and German	1
HAP [45]	3-point ordinal scale: ‘still doing, have stopped doing, never did’	102	Current	Contact author	5–10	No	No	English	1
ICECAP-A [46]	7-point best–worst scale	5	Not stated	Registration and contact author	≈1	No	No	Multiple languages including Chinese, Czech, Danish, Dutch, Spanish, French and German	1
ICQ [47]	4-point Likert scale	18	Not stated	Not stated	≈3	No	No	English	1
IPAQ [48]	Yes/no, time spent on activities	27	Recent, last 7 days	No charge	15–30	No	No	Native language	1
Katz ADL [49–51]	Dependent or independent	6	Past 2 weeks	Not stated	<2	Yes	No	English, Turkish	5
KDQOL [19]	Yes/no, 3-/5-/6-point Likert scale	134	Last 30 days	Available upon request to those measuring QOL in patients on dialysis	≈27	No	No^e^	Multiple including English, French, Japanese and Spanish	1
KDQOL-36 [20]	Yes/no, 3-/5-/6-point Likert scale	36	Current, past 4 weeks	No charge	≈10	No	No^e^	Multiple including English, French, Cantonese, Korean, Spanish and Turkish	5
KDQOL-SF [21]	Yes/no, 3-/5-/6-/10-point Likert scale	80	Current, past 4 weeks	No charge	16	No	No^e^	>10 languages including English, Chinese and Korean	24
KDQ [22]	7-point Likert scale	26	Past 2 weeks	Not stated	≈15	No	No^e^	English	1
K-ADL [52]	3-point Likert scale	7	Not stated	Not stated	<2	Yes	No	English, Korean	1
K-IADL [52]	3-point Likert scale	10	Not stated	Not stated	≈2	Yes	No	English, Korean	1
LLFDI [53, 54]	5-point Likert scale	48	Not stated	Contact author	≈10	No	No	Available in multiple languages	1
Lawton and Brody's IADL Scale [55]	3-/4-/5-point scale	8	Not stated	Not stated	10–15	Yes	No	English, Japanese	2
Lawton IADL [56]	3-/4-/5-point scale	8	Not stated	Not stated	10–15	Yes	No	English, Chinese, Turkish, Spanish, Hong Kong Chinese, Korean, Persian	4
LASA [57]	9-point Likert scale	5	Not stated	Not stated	≈1	No	No	English, German	2
MOS [58]	Not stated	116	Not stated	Not stated	≈24	No	No	English	1
NQOL [59]	7-point Likert scale	29	Past 2 weeks	Not stated	<12	No	No	Not stated	1
SF-6D [60]	4-/5-/6-point ordinal scale	6	Current	No charge for non-commercial use. License fee for commercial use	<2	No	No	Multiple	2
SF-12 [61]	Yes/no, 3-/5-/6-point Likert scale	12	Past 4 weeks	License fee	≈2	No	No	Multiple	3
SF-36 [62]	Yes/no, 3-/5-/6-point Likert scale	36	Current, past 4 weeks, 3 months	Free for non-commercial use/annual licence fee	5–10	No	No	170 languages including English, Sinhala, Chinese, Arabic, German and Dutch	39
SIP [63]	‘Check those that apply’	136	Current	Contact the Medical Outcomes Trust	20–30	No	No	Not stated	1
WHOQOL-BREF [12]	5-point Likert scale	26	Current, past 2 weeks	No charge	≈5	No	No	Multiple	8
WHOQOL-OLD FACETS [64]	5-point Likert scale	24	Not stated	Fill in form	≈5	No	No	>20 languages	1
WPAI [65]	Yes/no, number of hours, 11-point Likert scale	6	Past 7 days	No charge	<2	No	No	English and >140 other languages	3
WPAI-SHP [65]	Yes/no, number of hours, 11-point Likert scale	6	Past 7 days	Contact author	<2	No	No	English and 38 other translations	1
WSAS [28]	9-point Likert scale	5	Not stated	Not stated	≈1	Yes	No	English, German and British Sign Language	2
Author developed measures									
FS developed by Lin [18]	4-point Likert scale	26	Past month	Not stated	≈5	No	No^e^	Not stated	1
Hao *et al.* [66]	Five check boxes—very much/somewhat/a little bit	5	Not stated	Not stated	<2	No	No	English	1
Martini bADLS [67]	3-point Likert scale (dependent, semi-dependent, independent)	6	Not stated	Not stated	≈1	Yes	No	English	1
McClellan *et al.* [68]	‘How would you compare your activity level to others your age? Would you say you are less active, same as others your age, more active, don't know/not sure?’ Individuals were defined as less active if they said yes to the first category	1	Not stated	Not stated	≈1	No	No	English	1
Wang *et al.* BADL [69]	Assumed 10-point Likert scale. Limited data available	10	Data unavailable	Not stated	<2	Yes	No	Not stated	1
Wang *et al.* IADL [69]	Assumed 4-point Likert scale. Limited data available	6	Data unavailable	Not stated	≈1	Yes	No	Not stated	1

^a^Where time completion data were unavailable, authors estimated based on 12 seconds per item.

^b^CKD not requiring KRT.

^c^Language availability not necessarily validated in another language.

^d^Limited data available.

^e^Developed for dialysis and/or transplant.

15D: 15 dimensions; BAI: Modified Barthel Activities of Daily Living Index; Chula ADL: Chula Activities of Daily Living Index; DASI: Duke Activity Status Index; FACIT-SP: Functional Assessment of Chronic Illness Therapy – Spiritual Well-Being; HAP: Human Activity Profile; ICECAP-A: ICEpop CAPability measure for Adults; ICQ: Illness Cognition Questionnaire; IPAQ: International Physical Activity Questionnaire (long form); ADL: Activities of Daily Living; K-ADL: Korean Activities of Daily Living; K-IADL: Korean Instrumental Activities of Daily Living; LLFDI: Late Life Function and Disability Instrument; LASA: Linear Analog Scale Assessment; MOS: Medical Outcomes Study; NQOL: Nutrition Quality of Life; SF-12: 12-item Short Form; SIP: Sickness Impact Profile; WHOQOL-OLD FACETS: World Health Organization Quality of Life for Older People covering six facets; WPAI: Work Productivity and Activity Impairment; WPAI-SHP: Work Productivity and Activity Impairment – Specific Health Problem; WSAS: Work and Social Adjustment Scale; FS developed by Lin; bADLS: Scale of Basic Activities of Daily Living; BADL: Basic Activities of Daily Living.

No measures were specifically designed to assess life participation in CKD patients not requiring KRT. Twelve (29%) were designed specifically to assess the ability to participate in life activities (e.g. leisure, family, work, social), while 29 (71%) measures assessed broader constructs such as quality of life, health status, general well-being and psychological well-being, with a subscale or questions relevant to life participation.

None of the measures were developed specifically for CKD not requiring KRT. Five (12%) were developed for use in patients receiving dialysis (author developed fatigue scale (FS) by Lin [18]) and/or with a kidney transplant {Kidney Disease Quality of Life (KDQOL) [19], 36-item KDQOL (KDQOL-36) [20], KDQOL-SF [21] and Kidney Disease Questionnaire (KDQ) [22]}. Thirty-six (88%) were developed for the general population or conditions other than CKD.

The time taken to complete each measure ranged from 2 to 30 min. The number of items in each measure ranged from 1 to 136. Twelve (29%) measures asked patients to report their current life participation. The recall period ranged from ‘currently’ to the ‘last 3 months’. Thirteen (32%) measures were free of charge, 2 (5%) incurred a licensing fee and 7 (17%) required contact with the author. The remaining 19 (46%) were unclear about licensing. At least 26 (67%) measures have been translated and are available in a language other than English.

### Content of measures

All measures assessed obligatory activities, of which 29 (71%) measures assessed both obligatory and non-obligatory measures and 12 (29%) assessed only obligatory activities. No measure assessed only non-obligatory activities. Six (15%) measures stated specific activities, with the specific activities listed in each measure varying across the different measures. Examples of specific obligatory activities included in the measures were buying groceries, washing oneself, study or work, while specific non-obligatory activities included sport and socializing. Thirty-five (85%) measures asked more general questions about participation, e.g. ability to complete usual activities and ‘things they have to do’. A detailed summary of the activities assessed in each category is provided in Tables 2 and 3.

**Table 2:** Dimensions of life participation assessed by each measure.

		Non-	
Measure	Obligatory	obligatory	Types of activities
15D [36]	**•**	**•**	Walking (indoors, outdoors), read, sleep, eat, speak, housework, work outside the home, social interactions (friends, family, meetings, recreation, leisure)
Modified BAI [37, 38]	**•**		Daily activities (e.g. grooming, toilet use, mobility, transfer chair to bed, dressing, stairs, bathing)
Chula ADL [39]	**•**		Daily activities (walking outdoors, cooking, using public transportation, doing heavy housework, exchanging money)
DASI [40]	**•**	**•**	Self-care (dressing, bathing, eating, toilet), daily activities (walking, climbing stairs/hill, housework, dishes, lifting heavy objects, carrying groceries, vacuuming, yard work), sex life, recreational/leisure activities (golf, throwing the ball, tennis), strenuous sports (swimming, basketball), running
EQ-5D [41, 42]	**•**	**•**	Mobility (walking), self-care (washing, dressing), usual activities (work, study housework, family, leisure activities)
EQ-5D-3L [41]	**•**	**•**	Mobility (walking), self-care (washing, dressing), usual activities (work, study housework, family, leisure activities)
EQ-5D-5L [42]	**•**	**•**	Mobility (walking), self-care (washing, dressing), usual activities (work, study housework, family, leisure activities)
FACT-An [43]	**•**	**•**	Daily activities (time spent in bed, meeting family needs, housework, usual activities), work outside the home, social activities (friends, family, relationships, sex life, leisure), sleep, walking
FACIT-SP [44]	**•**	**•**	Daily activities (time spent in bed, meeting family needs, housework), work outside the home, social activities (friends, family, relationships, sex life, leisure), sleep
HAP [45]	**•**	**•**	Cooking meals, putting on shoes, climbing steps, sweeping, walking, mowing the lawn, dining at a restaurant, dancing
ICECAP-A [46]	**•**	**•**	Settled and secure, social (love, friends, support) enjoyment, pleasure, independence, achievements and progress
ICQ [47]	**•**	**•**	Daily activities, enjoyment/leisure activities, limitations
IPAQ [48]	**•**	**•**	Work outside the home (paid or unpaid), daily activities (e.g. house maintenance, shovelling snow, chopping wood, gardening, cleaning, scrubbing floors), travelling, transportation, recreation/leisure and sports (e.g. walking, swimming, aerobics, double tennis, bicycling)
Katz ADL [49, 50, 51]	**•**		Daily activities (bathing, dressing, toileting, transferring, continence, feeding)
KDQOL [19]	**•**	**•**	Daily activities (e.g. housework, moving a table, pushing a vacuum cleaner, carrying groceries, climbing stairs, lifting heavy objects, bathing or dressing, bending, kneeling or stooping), sport (e.g. running, participating in strenuous sports, walking, bowling or playing golf), social activities (e.g. friends, family), work outside the home, sex life, travel, sleep
KDQOL-36 [20]	**•**	**•**	Daily activities (e.g. housework, moving a table, pushing a vacuum cleaner, carrying groceries, climbing stairs), bowling, playing golf, social activities (e.g. friends, family), work outside the home, sex life, travel
KDQOL-SF [21]	**•**	**•**	Daily activities (e.g. housework, moving a table, pushing a vacuum cleaner, carrying groceries, climbing stairs, lifting heavy objects, bathing or dressing, bending, kneeling or stooping), sport (e.g. running, participating in strenuous sports, walking, bowling or playing golf), social activities (e.g. friends, family), work outside the home, sex life, travel, sleep
KDQ [22]	**•**		Housework
K-ADL [52]	**•**		Daily activities (dressing, washing face and hands, bathing, eating, transfer, toileting, continence)
K-IADL [52]	**•**		Daily activities (decorating, housework, preparing meals, laundry, outgoing for a short distance, using transportation, shopping, handling money, using telephone, taking medicine)
LLFDI [53, 54]	**•**	**•**	Social activities (friends, interaction, volunteering, help others), activity (walking, hiking, exercise), daily activities (climbing stairs, lifting, catching bus, bend over, standing up from couch, dressing, using utensils, dishes, housework, errands)
Lawton and Brody's IADL Scale [55]	**•**		Daily activities (using the phone, shopping, preparing food, housekeeping, doing laundry, using transportation, handling medications, handling finances)
Lawton IADL [56]	**•**		Daily activities (using the phone, shopping, preparing food, housekeeping, doing laundry, using transportation, handling medications, handling finances)
LASA [57]	**•**	**•**	Social activities (friends, interaction, pleasure, relationships), physical well-being, fatigue
MOS [58]	**•**	**•**	Daily activities (e.g. housework, moving a table, pushing a vacuum cleaner, carrying groceries, climbing stairs, lifting heavy objects, bathing or dressing, bending, kneeling or stooping), travel, mobility limitations, assistance, ability to work (outside the home and housework), recreational/leisure activities, enjoyment, walking/movement, social activities (e.g. friends, family)
NQOL [59]	**•**	**•**	Social activities (friends, interaction, pleasure, relationships), daily activities (grocery shopping, cooking)
SF-6D [60]	**•**	**•**	Daily activities (e.g. housework, bathing, dressing), social activities, vigorous activities, work outside the home
SF-12 [61]	**•**	**•**	Daily activities (e.g. housework, moving a table, pushing a vacuum cleaner, climbing stairs, bowling, playing golf), social activities (e.g. friends, family), work outside the home
SF-36 [62]	**•**	**•**	Daily activities (e.g. housework, moving a table, pushing a vacuum cleaner, carrying groceries, climbing stairs, lifting heavy objects, bathing or dressing, bending, kneeling or stooping), social activities (e.g. friends, family), work outside the home
SIP [63]	**•**	**•**	Usual daily activities, household management (bills, banking), mobility, body movement, leisure and recreation, decision making, social (family, friends, caring for children), walking, limitations
WHOQOL-BREF [12]	**•**	**•**	Daily living activities/necessary tasks, leisure activities, mobility, sleep, work outside the home, social (relationships, sex life, friends)
WHOQOL-OLD [64]	**•**	**•**	Daily activities, interaction, decision making, social participation, engagement and participation in activities, relationships
WPAI [65]	**•**	**•**	Work, regular activities (housework, shopping, childcare, exercising, studying)
WPAI-SHP [65]	**•**	**•**	Work, regular activities (housework, shopping, childcare, exercising, studying)
WSAS [28]	**•**	**•**	Ability to work, home management (cleaning, tidying, shopping, bills, caring for children, cooking), social leisure activities (parties, outings, visits, relationships, home entertaining), private leisure (reading, gardening, sewing, walking), close relationships
Author developed measures			
FS developed by Lin [18]	**•**		Physical ability, motivation, mental ability, daily activities
Hao et al. [66]	**•**	**•**	Meeting family needs, enjoyment, ability to work (outside the home and housework), recreation/leisure, friends, family, relationships, sex life, lack of energy, sleep, time in bed, ill, nausea, side effects, quality of life, worry, coping, sad, support
Martini bADLS [67]	**•**		Daily activities (using the phone, shopping, preparing food, housekeeping, doing laundry, using transportation, handling medications, handling finances)
McClellan [68]	**•**	**•**	Daily activities, physical activity
Wang et al. BADL [69]	**•**		Daily activities (e.g. grooming, toilet use, mobility, transfer chair to bed, dressing, stairs, bathing)
Wang IADL [69]	**•**		Daily activities (using the phone, shopping, preparing food, housekeeping, doing laundry, using transportation, handling medications, handling finances)

15D: 15 Dimensions; BAI: Modified Barthel Activities of Daily Living Index; Chula ADL: Chula Activities of Daily Living Index; DASI: Duke Activity Status Index; FACIT-SP: Functional Assessment of Chronic Illness Therapy – Spiritual Well-Being; HAP: Human Activity Profile; ICECAP-A: ICEpop CAPability measure for Adults; ICQ: Illness Cognition Questionnaire; IPAQ: International Physical Activity Questionnaire (long form); ADL: Activities of Daily Living; K-ADL: Korean Activities of Daily Living; K-IADL: Korean Instrumental Activities of Daily Living; LLFDI: Late Life Function and Disability Instrument; LASA: Linear Analog Scale Assessment; MOS: Medical Outcomes Study; NQOL: Nutrition Quality of Life; SF-12: 12-item Short Form; SIP: Sickness Impact Profile; WHOQOL-OLD FACETS: World Health Organization Quality of Life for Older People covering six facets; WPAI: Work Productivity and Activity Impairment; WPAI-SHP: Work Productivity and Activity Impairment – Specific Health Problem; bADLS: Scale of Basic Activities of Daily Living; BADL: Basic Activities of Daily Living.

**Table 3:** Dimensions of life participation assessed by each measure: A visual.

			Physical activities			Social activities								
Measure	Obligatory	Non-obligatory	Running	Walking	Sports	Family	Friends	Relationships/sex life	Leisure activities	School/work	Household	Daily activities	Travel	Other
15D [36]	**•**	**•**		**•**		**•**	**•**		**•**	**•**	**•**	**•**		
Modified BAI [37, 38]	**•**	**•**										**•**		
Chula ADL [39]	**•**	**•**		**•**							**•**	**•**		
DASI [40]	**•**	**•**	**•**	**•**	**•**			**•**	**•**		**•**	**•**		
EQ-5D [41, 42]	**•**	**•**		**•**		**•**	**•**		**•**	**•**		**•**		
EQ-5D-3L [41]	**•**	**•**		**•**		**•**	**•**		**•**	**•**		**•**		
EQ-5D-5L [42]	**•**	**•**		**•**		**•**	**•**		**•**	**•**		**•**		
FACT-An [43]	**•**	**•**		**•**		**•**	**•**	**•**	**•**	**•**	**•**	**•**		
FACIT-SP [44]	**•**	**•**				**•**	**•**	**•**	**•**	**•**	**•**	**•**		
HAP [45]	**•**	**•**		**•**	**•**						**•**	**•**		
ICECAP-A [46]	**•**	**•**					**•**	**•**	**•**					Achievements and settled
ICQ [47]	**•**								**•**			**•**		
IPAQ [48]	**•**			**•**	**•**				**•**	**•**	**•**	**•**	**•**	
Katz ADL [49, 50, 51]	**•**											**•**		
KDQOL [19]	**•**		**•**	**•**	**•**	**•**	**•**	**•**		**•**	**•**	**•**	**•**	
KDQOL-36 [20]	**•**				**•**	**•**	**•**	**•**		**•**	**•**		**•**	
KDQOL-SF [21]	**•**		**•**	**•**	**•**	**•**	**•**	**•**		**•**	**•**	**•**	**•**	
KDQ [22]	**•**										**•**			
K-ADL [52]	**•**	**•**										**•**		
K-IADL [52]	**•**	**•**									**•**	**•**		
LLFDI [53, 54]	**•**	**•**		**•**	**•**		**•**				**•**	**•**		
Lawton and Brody's IADL Scale [55]	**•**	**•**									**•**	**•**		
Lawton IADL [56]	**•**	**•**									**•**	**•**		
LASA [57]	**•**	**•**					**•**	**•**						Wellbeing
MOS [58]	**•**	**•**		**•**		**•**	**•**		**•**	**•**	**•**	**•**	**•**	
NQOL [59]	**•**	**•**					**•**	**•**			**•**			
SF-6D [60]	**•**	**•**					**•**			**•**	**•**	**•**		Vigorous activities
SF-12 [61]	**•**	**•**			**•**	**•**	**•**			**•**	**•**			
SF-36 [62]	**•**	**•**				**•**	**•**			**•**	**•**	**•**		
SIP [63]	**•**	**•**		**•**		**•**	**•**		**•**		**•**	**•**		
WHOQOL-BREF [12]	**•**	**•**					**•**	**•**	**•**	**•**	**•**	**•**		
WHOQOL-OLD [64]	**•**	**•**				**•**	**•**	**•**				**•**		
WPAI [65]	**•**	**•**			**•**					**•**	**•**	**•**		
WPAI-SHP [65]	**•**	**•**			**•**					**•**	**•**	**•**		
WSAS [28]	**•**	**•**				**•**	**•**	**•**	**•**	**•**	**•**	**•**		
Author developed measures														
FS developed by Lin [18]	**•**											**•**		Physical and mental mobility, motivation
Hao et al. [66]	**•**	**•**				**•**	**•**	**•**	**•**	**•**	**•**	**•**		Enjoyment
Martini et al. bADLS [67]	**•**										**•**	**•**		
McClellan [68]	**•**	**•**										**•**		Physical activity
Wang et al. BADL [69]	**•**											**•**		
Wang et al. IADL [69]	**•**										**•**	**•**		

15D: 15 dimensions; BAI: Modified Barthel Activities of Daily Living Index; Chula ADL: Chula Activities of Daily Living Index; DASI: Duke Activity Status Index; FACIT-SP: Functional Assessment of Chronic Illness Therapy – Spiritual Well-Being; HAP: Human Activity Profile; ICECAP-A: ICEpop CAPability measure for Adults; ICQ: Illness Cognition Questionnaire; IPAQ: International Physical Activity Questionnaire (long form); ADL: Activities of Daily Living; K-ADL: Korean Activities of Daily Living; K-IADL: Korean Instrumental Activities of Daily Living; LLFDI: Late Life Function and Disability Instrument; IADL: Instrumental Activities of Daily Living; LASA: Linear Analog Scale Assessment; MOS: Medical Outcomes Study; NQOL: Nutrition Quality of Life; SF-12: 12-item Short Form; SIP: Sickness Impact Profile; WHOQOL-OLD FACETS: World Health Organization Quality of Life for Older People covering six facets; WPAI: Work Productivity and Activity Impairment; WPAI-SHP: Work Productivity and Activity Impairment – Specific Health Problem; bADLS: Scale of Basic Activities of Daily Living; BADL: Basic Activities of Daily Living.

### Psychometric properties

Of the 41 measures, only 4 have been validated in the CKD population. A summary of the data on the psychometric properties of the measures is provided in Table 4. The validation data and psychometric properties assessed for each of the measures were highly variable and no measure provided information across all psychometric domains.

**Table 4:** Summary of validation data of psychometric properties of measures that have been used to assess life participation in CKD.

Measure/psychometric properties	Content validity	Convergent validity	Known groups validity	Responsiveness	Test–retest reliability	Internal consistency	Structural validity	Measurement error	Criterion validity	Cross-cultural validity	Total (N = 11)
EQ-5D-3L		**•**	**•**			**•**					**3**
FACT-An		**•**	**•**	**•**	**•**	**•**					**5**
SF-6D		**•**									**1**
SF-36		**•**	**•**	**•**	**•**	**•**					**5**

EQ-5D-3L: EuroQol 5-dimension 3-level; FACT-An: Functional Assessment of Cancer Therapy – Anemia; SF-6D; Short Form 6-dimension; SF-36: Short Form 36-dimension.

The EuroQol 5-dimension 3-level (EQ-5D-3L) measure indicated good internal consistency with a Cronbach's alpha of 0.834 [23]. Both the EQ-5D-3L and SF-36 were able to differentiate between depressed/not depressed and with/without psychological distress groups [23].

The Functional Assessment of Cancer Therapy - Anemia (FACT-An) indicated high internal consistency and test–retest reliability with Cronbach's alpha ranging from 0.79 to 0.95 and the intraclass correlation coefficient ranging from 0.72 to 0.88 [24]. The FACT-An fatigue and anaemia domains showed strong correlations with the SF-36 vitality domain (Pearson's *r* = 0.76 and *r* = 0.77, respectively) [24].

The 6-dimension Short Form (SF-6D) indicated varying convergent validity, with the pain domain strongly correlating with the role and control domains of ICEpop CAPability measure for Older people (ICECAP-O) with a Pearson's *r* coefficient of 0.51 and 0.53, respectively; however the role domain of ICECAP-O was weakly correlated with the SF-6D role and social functioning domain (0.28 and 0.34, respectively) [25].

The SF-36 and FACT-An demonstrated responsiveness, with small improvements (relative to baseline) seen in all SF-36 domains. The largest gains were seen in the dialysis group and only the vitality domain reached this cut-off (a >3-point increase by week 9 or week 17 of the trial) in the non-dialysis group. Internal consistency was high in key life participation domains of social functioning, role physical and physical functioning, reporting Cronbach's alpha of 0.76, 0.93 and 0.90, respectively [26].

## DISCUSSION

Life participation has been identified as a critically important outcome in CKD patients not requiring KRT and their caregivers; however, it is inconsistently reported in trials and observational studies, with 41 different measures used across 114 studies. Most of the measures assessed a broader construct (quality of life), with at least one item relevant to life participation rather than life participation as a specific construct. The measures varied widely in terms of completion time, response format, number of items, recall period, cost, content and availability of psychometric data. Data on the characteristics and psychometric properties in CKD populations for many of the measures were very limited.

The most common measures used (SF-36, KDQOL-SF, WHOQOL-BREF) assessed life participation with a single question/item within broader constructs of quality of life. There were nine measures that specifically assessed life participation (or activities of daily living), with the most frequent measures being the Katz Activities of Daily Living (ADL), Lawton Instrumental Activities of Daily Living (IADL), Lawton and Brody IADL and Work and Social Adjustment Scale (WSAS). Some measures asked patients to assess their life participation in general, while other measures asked about specific activities. These specific activities included obligatory activities (e.g. walking, washing) and non-obligatory activities (e.g. sport, social and leisure activities). These measures are not feasible to implement in trials due to the length of the measures, are not specific to life participation and are not validated in CKD patients not requiring KRT. By routinely measuring someone's ability to participate in life, it provides an opportunity to maintain the ability to participate in life as well as in prevention and intervention, which are critical in delaying progression [7]. However, trials that do not incorporate PROMs are at risk of undermining the relevance, reliability and value of the research, consequently limiting their impact in policy and practice [27].

The measures identified in our review were designed to be used in the general population or in patients receiving dialysis or with a kidney transplant. No measure was specifically designed for CKD patients not requiring KRT. Only four measures (EQ-5D-3L, FACT-An, SF-6D, SF-36) have been validated in CKD patients not requiring KRT. Of these four measures, convergent validity was the most common psychometric property that has been assessed, followed by validity and internal consistency of known groups. Measurement error, criterion, cross-cultural and structural validity were not found for any measure. The lack of assessments for psychometric robustness of these measures in CKD patients not requiring KRT therapy leaves the validity, responsiveness and reliability unknown.

At least 26 (67%) of the measures have been translated to languages other than English; however, there may be culturally appropriate adaptations, but this was not the aim of our search. Availability of measures in multiple languages is key to transferability in non-English-speaking populations. Translations and cultural adaptions are required to ensure valid measurement of life participation in non-English-speaking individuals [29]. The availability of appropriately translated PROMs for varying cultures and languages will help ensure generalizability, limiting missing data and sample attrition [29].

Life participation has been identified as a core outcome in peritoneal dialysis [30], transplant [31] and for children and adolescents with CKD [32], but remains infrequently and inconsistently measured in research. In trials and observational studies in kidney transplant recipients, 29 measures were used to assess life participation, with the most frequently used measures being the SF-36 [155 studies (67%)], KDQOL-SF [36 studies (16%)] and EQ-5D [12 studies (5%)] [10]. More than half of the 29 measures assessed the broader construct of quality of life and 13 measures (45%) included specific questions regarding life participation [10]. Similarly in trials and observational studies in patients receiving peritoneal dialysis, 42 measures were used, with the most frequently used measures being the SF-36 [122 studies (41%)], KDQOL-SF [86 studies (29%)] and EQ-5D [27 studies (9%)] [33]. Most measures were designed to assess quality of life [27 measures (64%)] rather than life participation [15 measures (36%)] specifically [33].

According to the WHO’s International Classification of Functioning, Disability and Health, life participation is a multifaceted construct that includes three components with 10 domains: body function, environmental factors and activities and participation [34]. Activities and participation include the domains of mobility, self-care, major life areas, community and social and civic life [34]. Consequently, these domains should be considered when measuring life participation in patients with CKD. The measures identified in this review broadly capture these domains.

Our review included a comprehensive search to identify all measures of life participation that had been reported in trials and observational studies in patients with CKD. However, it is possible that there are other measures that may be potentially appropriate for use in patients with CKD.

There is a need to establish a standardized and validated measure of life participation for CKD patients not requiring KRT for routine use as a core outcome measure in clinical trials. Through the Standardised Outcomes in Nephrology (SONG) initiative, life participation has been established in >1000 patients, caregivers and health professionals from >70 countries as a core outcome to be reported in trials in CKD patients not requiring KRT [11]. Efforts are currently under way to establish a meaningful and validated core outcome measure for life participation for CKD patients that is feasible to implement and both short yet broad enough to capture an individual's priorities and circumstances. This will include consideration of the recently developed SONG Life Participation measure developed for kidney transplant recipients [35], which includes five items on a 5-point response scale. These domains include leisure activities (e.g. exercise, hobbies, travel), family activities, work (e.g. employment, housework, study) and social activities (friends/others) [35]. Validating a PROM for life participation in CKD patients not requiring KRT will involve international multistakeholder consensus workshops and pilot and validation studies. Further work will also be undertaken to ensure language and cross-cultural validity of the final measure.

There is increasing recognition of the need to include core outcomes and measures in all trials to ensure that the evidence generated is meaningful to patients. Developing and implementing a content-relevant and validated core outcome measure for life participation in CKD patients not yet requiring KRT in trials will ensure evidence is available to better support shared decision-making and inform interventions to improve the core patient-important outcome of life participation.

## Supplementary Material

sfae341_Supplemental_File

## Data Availability

The data underlying this article are available in the article and in the online supplementary material.
