# Supplementary material for: Patient-reported outcome measures for life participation in patients with chronic kidney disease: a systematic review
Source: Clin Kidney J. 2024 Nov 12;18(1):sfae341. doi: 10.1093/ckj/sfae341 (PMC11730184; doi:10.1093/ckj/sfae341)
Supplement: sfae341_Supplemental_File [file sfae341_supplemental_file.docx]

**SUPPLEMENTARY MATERIAL: Patient-reported outcome measures for life participation in patients with chronic kidney disease: a systematic review**

**S1. Search strategies**

**Embase 1974 to February 07, 2023**

| Searches |
| --- |
| 1. exp chronic kidney failure/ |
| 1. chronic kidney disease.tw. |
| 1. pre-dialysis.tw. |
| 1. or/1-3 |
| 1. exp clinical trial/ or exp controlled study/ or exp randomized controlled trial/ |
| 1. trial.tw. |
| 1. exp epidemiology/ |
| 1. case control.tw. |
| 1. cohort study.tw. |
| 1. exp observational study/ |
| 1. exp prospective study/ |
| 1. exp cross-sectional study/ |
| 1. or/5-12 |
| 1. exp “quality of life”/ |
| 1. QOL.tw. |
| 1. exp Sickness Impact Profile/ |
| 1. exp daily life activity/ |
| 1. activities of life.tw. |
| 1. activities of liv$.tw. |
| 1. exp leisure/ |
| 1. exp travel/ |
| 1. exp work capacity/ or exp work/ or exp return to work/ |
| 1. exp school/ |
| 1. exp employment/ |
| 1. life participation.tw. |
| 1. life involvement.tw. |
| 1. daily activit$.tw. |
| 1. exp fatigue/ |
| 1. fatigue.tw. |
| 1. exp chronic fatigue syndrome/ |
| 1. (weary or weariness or exhaust$).tw. |
| 1. tired$.tw. |
| 1. lethargy$.tw. |
| 1. (energy$ or vigor$ or vigour$).tw. |
| 1. exp Sleep/ |
| 1. sleep.tw. |
| 1. or/14-36 |
| 1. 4 and 13 and 37 |
| 1. exp short survey/ or exp health survey/ |
| 1. exp structured questionarie/ or exp questionnaire/ |
| 1. survey$.tw. |
| 1. questionnaire$.tw. |
| 1. exp patient-reported outcome/ |
| 1. patient-reported outcome$.tw. |
| 1. PROM$.tw. |
| 1. index$.tw. |
| 1. instrument.tw. |
| 1. or/39-47 |
| 1. 38 and 48 |

**MEDLINE 1946 to February 07, 2023**

| Searches |
| --- |
| 1. exp chronic kidney disease/ |
| 1. chronic kidney disease.tw. |
| 1. exp Renal Insufficiency, Chronic/ or exp Kidney Failure, Chronic/ |
| 1. pre-dialysis.tw. |
| 1. or/1-4 |
| 1. Randomized controlled trial.pt. |
| 1. controlled clinical trial.pt. |
| 1. exp Clinical Trial/ |
| 1. exp epidemiologic studies/ |
| 1. case control.tw. |
| 1. (cohort adj stud*).tw. |
| 1. exp Observational Study/ |
| 1. exp Prospective Studies/ |
| 1. exp Cross-Sectional Studies/ |
| 1. or/6-14 |
| 1. exp quality of life/ |
| 1. QOL.tw. |
| 1. exp sickness impact profile/ |
| 1. exp activities of daily living/ |
| 1. exp leisure activities/ |
| 1. exp travel/ |
| 1. exp work/ |
| 1. exp school/ |
| 1. activities of daily living$.tw. |
| 1. exp employment/ |
| 1. life participation.tw. |
| 1. life involvement.tw. |
| 1. daily activitie$.tw. |
| 1. exp fatigue/ |
| 1. fatigue.tw. |
| 1. chronic fatigue syndrome.tw. |
| 1. (weary or weariness or exhaust$).tw. |
| 1. tired$.tw. |
| 1. letharg$.tw. |
| 1. (energy$ or vigor$ or vigour$).tw. |
| 1. exp Sleep/ |
| 1. sleep.tw. |
| 1. exp Muscle Fatigue/ or exp Mental Fatigue/ |
| 1. or/16-38 |
| 1. 5 and 15 and 39 |
| 1. exp "Surveys and Questionnaires"/ |
| 1. (survey$ or questionnaire$).tw. |
| 1. exp Patient Outcome Assessment/ |
| 1. exp Patient Reported Outcome Measures/ |
| 1. PROM$.tw. |
| 1. patient-reported$.tw. |
| 1. exp Health Surveys/ |
| 1. (index$ or score$).tw. |
| 1. instrument$.tw. |
| 1. or/41-49 |
| 1. 40 and 50 |

**PsycINFO 1967 to 7^th^ February 2023**

| Searches |
| --- |
| 1. exp Kidney Diseases/ |
| 1. chronic kidney disease.tw. |
| 1. chronic kidney failure.tw. |
| 1. or/1-3 |
| 1. exp "Quality of Life"/ |
| 1. exp "Activities of Daily Living"/ |
| 1. daily living$.tw. |
| 1. exp Leisure Time/ or exp Recreation/ |
| 1. exp Traveling/ |
| 1. exp Work-Life Balance/ |
| 1. exp Schools/ |
| 1. study$.tw. |
| 1. exp Employment Status/ |
| 1. exp Participation/ |
| 1. life participat$.tw. |
| 1. life involvement$.tw. |
| 1. exp Chronic Fatigue Syndrome/ or exp Fatigue/ |
| 1. fatigue.tw. |
| 1. (weary or weariness or exhaust$).tw. |
| 1. tired$.tw. |
| 1. letharg$.tw. |
| 1. (energy$ or vigor$ or vigour$).tw. |
| 1. exp Sleep Deprivation/ or exp Sleep/ |
| 1. sleep$.tw. |
| 1. or/5-24 |
| 1. 4 and 25 |
| 1. exp Mail Surveys/ or exp Telephone Surveys/ or exp Surveys/ or exp Online Surveys/ |
| 1. survey$.tw. |
| 1. exp Questionnaires/ |
| 1. questionnaire$.tw. |
| 1. (index$ or score$).tw. |
| 1. instrument$.tw. |
| 1. exp Patient Reported Outcome Measures/ |
| 1. patient-reported$.tw. |
| 1. or/27-34 |
| 1. 26 and 35 |

**CINAHL**

| Search ID | Search terms |
| --- | --- |
| S1 | (MH "Kidney Failure, Chronic+") OR (MH "Renal Insufficiency, Chronic+") |
| S2 | (MH "Fatigue+") OR (MH "Fatigue Syndrome, Chronic") OR (MH "Mental Fatigue+") |
| S3 | (MH "Activities of Daily Living+") |
| S4 | S2 OR S3 |
| S5 | S1 AND S4 |

**S2. Characteristics of randomized, non-randomized and observational studies**

**Table a. Characteristics of randomized controlled trials**

| Study | N | Country | Intervention | Measure used |
| --- | --- | --- | --- | --- |
| Akizawa 2011 ^1^ | 322 | Japan | A high Hb group to receive darbepoetin alfa (DA) or a low Hb group to receive recombinant human erythropoietin (rHuEPO). | SF-36 |
| Alexander 2007 ^2^ | 81 | US | Receiving darbepoetin alfa in addition to conversative management for CKD. | SF-36, KDQOL, Katz ADL and Lawton IADL |
| Arnold 2017 ^3^ | 47 | Australia | Dietary potassium restriction protocol with the aim of keeping serum potassium level below 4.5 mEq/L. | SF-36 |
| Blakeman 2014 ^4^ | 436 | UK | Kidney information guidebook; a booklet and interactive website that tailored access to community resources; and telephone-guided help from a lay health worker. | MOS |
| Bohlke 2022 ^5^ | 150 | Brazil | 16 week physical training | SF-36 |
| Campbell 2008 ^6^ | 53 | Australia | Nutrition | KDQOL-SF |
| Greenwood 2022 ^7^ | 74 | UK | Intravenous iron therapy | WSAS |
| Hedayati 2017 ^8^ | 201 | US | Treatment with sertraline to improve depressive symptoms | WSAS, KDQOL-SF |
| Hirakata 2010 ^9^ | 171 | Japan | A higher Hb target (12–13 g/dl) by darbepoetin alfa (DPO) was compared with the conventional Hb target by epoetin alfa (EPO). | SF-36 |
| Lewis 2011 ^10^ | 4038 | US, Canada | Darbepoetin Alfa | SF-36 |
| Li 2020 ^11^ | 49 | Taiwan | Effectiveness of wearable devices, a health management platform, and social media. | KDQOL-SF |
| Magar 2017 ^12^ | 120 | Canada | Daily v monthly vitamin d supplementation | SF-36 |
| Mustata 2011 ^13^ | 20 | Canada | N/A | SF-36 |
| Provenzano 2005 ^14^ | 519 | US | 10,000 units (U) once weekly (QW), 20,000 U every two weeks (Q2W), 30,000 U every three weeks (Q3W) or 40,000 U every four weeks (Q4W). | LASA |
| Roger 2014 ^15^ | 51 | Australia, Canada | Darbepoetin alfa | SF-36, FACT-An |
| Rossi 2014 ^16^ | 96 | US | Guided exercise two times per week for 12 weeks. | RAND-36 |
| Shin 2022 ^17^ | 149 | Republic of Korea/South Korea | AST-120 | KDQOL-SF |
| Uchiyama 2021 ^18^ | 46 | Japan | Exercise group patients performed aerobic exercise at 40–60% peak heart rate thrice weekly and resistance training at 70% of one-repetition maximum twice weekly at home for 6 months. | KDQOL-SF |
| van Craenenbroeck 2015 ^19^ | 40 | Belgium | 3-month home-based aerobic training program consisted of 4 daily cycling sessions of 10 minutes each at a target heart rate, calculated as 90% of the heart rate achieved at the anaerobic threshold. | KDQOL-SF |
| Villar 2011 ^20^ | 89 | France | Group 1 patients were assigned to a subnormal Hb level range (110–129 g/l) and group 2 to a normal Hb level range (130–149 g/l). | SF-36 |

FACT-An: Functional Assessment of Cancer Therapy – Anemia; KDQOL: Kidney Disease Quality of Life; LASA: Linear Analog Scale Assessment; MOS: Medical Outcomes Study; SF-36: Short-Form 36-Item Questionnaire; WSAS: Work and Social Adjustment Scale.

**Table b. Characteristics of non-randomized controlled trials**

| Study | N | Country | Intervention | Measure used |
| --- | --- | --- | --- | --- |
| Guo 2022 ^21^ | 49 | China | Non-protein energy supplement | SF-36 |
| Kuo 2015 ^22^ | 60 | Taiwan | CABG | WHOQOL-BREF |
| Lefebvre 2006 ^23^ | 1326 | US, Canada | Once-weekly (QW) dosing of epoetin alfa for the treatment of anemia in non-dialysis CKD patients | LASA, KDQ |

KDQ: Kidney Disease Questionnaire; LASA: Linear Analog Scale Assessment; SF-36: Short-Form 36-Item Questionnaire; WHOQOL-BREF: World Health Organisation Quality of Life Brief Version.

**Table c. Characteristics of observational studies**

| Study | N | Country | Intervention | Measure used |
| --- | --- | --- | --- | --- |
| Abeywickrama 2020 ^24^ | 120 | Sir Lanka | N/A | KDQOL-SF |
| Adame Perez 2019 ^25^ | 41 | Canada | N/A | SF-36 |
| Aggarwal 2016 ^26^ | 200 | India | N/A | SF-36 |
| Alshelleh 2022 ^27^ | 103 | Jordan | N/A | WHOQOL-BREF |
| Andrew 2020 ^28^ | 142 | India | N/A | WHOQOL-BREF |
| Arnold 2022 ^29^ | 109 | Australia | N/A | SF-36 |
| Baddour 2019 ^30^ | 271 | US | N/A | Katz ADL, Lawton IADL |
| Bagasha 2021 ^31^ | 364 | Uganda | N/A | KDQOL-SF |
| Belkin 2021 ^32^ | 350 | US | N/A | Lawton ADL |
| Bonner 2013 ^33^ | 28 | Australia | N/A | SF-36, HAP |
| Bowling 2022 ^34^ | 223 | US | N/A | Katz ADL, Lawton |
| Busa 2022 ^35^ | 1063 | UK | N/A | EQ-5D-5L, ICECAP-A |
| Chesnaye 2022 ^36^ | 1421 | Germany, Italy, The Netherlands, Poland, Sweden, and the UK. | N/A | SF-36 |
| Chin 2014 ^37^ | 984 | Korea | N/A | K-ADL, K-IADL |
| Chin 2008 ^38^ | 944 | Korea | N/A | SF-36 |
| Covic 2017 ^39^ | 1993 | France, Germany, Italy, Spain, and the UK | N/A | WPAI |
| Davison 2009 ^40^ | 185 | Canada | N/A | SF-6D |
| de Pina Pereira 2017 ^41^ | 35 | Brazil | N/A | WHOQOL-BREF DOMAINS, WHOQOL-OLD FACETS |
| Delgado 2009 ^42^ | 293 | Colombia | N/A | SF-36 |
| Erez 2016 ^43^ | 74 | England | N/A | SF-36 |
| Eriksson 2016 ^44^ | 2898 | France, Germany, Italy, Spain and the UK | N/A | EQ-5D-3L, WPAI |
| Farag 2011 ^45^ | 69 | US | N/A | KDQOL-SF |
| Feng 2013 ^46^ | 362 | Singapore | N/A | SF-12 |
| Finkelstein 2009 ^47^ | 1186 | North America | N/A | KDQOL-SF |
| Finkelstein 2018 ^48^ | 204 | US | N/A | SF-36 |
| Fraser 2020 ^49^ | 1008 | England | N/A | EQ-5D-5L |
| Guedes 2021 ^50^ | 2513 | Brazil, France, US | N/A | KDQOL-36 |
| Gunawardena 2020 ^51^ | 1079 | Sir Lanka | N/A | KDQOL-SF |
| Gyamlani 2011 ^52^ | 71 | US | N/A | SF-36 |
| Hakamaki 2021 ^53^ | 210 | Finland | N/A | KDQOL-SF |
| Hamilton 2006 ^54^ | 34 | US | N/A | SF-36 |
| Hanson 2009 ^55^ | 79 | US | N/A | KDQOL-SF |
| Hao 2021 ^56^ | 456 | China | N/A | Hao et al |
| Ho 2022 ^57^ | 242 | Taiwan | N/A | FS was developed by Lin et al. |
| Hoshino 2020 ^58^ | 2465 | Brazil, France, Germany, Japan, and the US | N/A | KDQOL-36 |
| Hussain 2019 ^59^ | 323 | India | N/A | EQ-5D-3L |
| Jalal 2022 ^60^ | 180 | Saudi Arabia | N/A | KDQOL-SF |
| Jesky 2016 ^61^ | 745 | UK | N/A | EQ-5D-3L |
| Jhamb 2014 ^62^ | 173 | US | N/A | SF-36 |
| Kaltsouda 2011 ^63^ | 98 | Greece | N/A | SF-36 |
| Kefale 2019 ^64^ | 256 | Ethiopia | N/A | SF-36 |
| Kharshid 2020 ^65^ | 526 | Malaysia | N/A | SF-36 (RAND-36) |
| Kim 2023 ^66^ | 970 | Korea | N/A | KDQOL-SF |
| Klang 1998 ^67^ | 56 | Sweeden | N/A | SIP |
| Korevaar 2000 ^68^ | 301 | The Netherlands | N/A | SF-36, EQ-5D-3L (EuroQol-3) |
| Kosmadakis 2012 ^69^ | 32 | UK | N/A | FACIT-SP |
| Krishnasamy 2016 ^70^ | 108 | Australia | N/A | SF-12 |
| Kularatna 2019 ^71^ | 1096 | Sir Lanka | N/A | EQ-5D-3L, SF-6D |
| Kusek 2002 ^72^ | 1094 | US | N/A | SF-36 |
| Lee 2021 ^73^ | 1268 | US | N/A | Lawton and Brody’s Instrumental ADL Scale, Katz Index of Independence in ADL, KDQOL and ADL items |
| Lee 2013 ^74^ | 208 | South Korea | N/A | WHOQOL-BREF |
| Lemos 2015 ^75^ | 170 | Brazil | N/A | SF-36 |
| Lidgard 2022 ^76^ | 3910 | US | N/A | KDQOL-36 |
| Lin 2022 ^77^ | 497 | Taiwan | N/A | SF-12 |
| Mansur 2014 ^78^ | 61 | Brazil | N/A | SF-36 |
| Martini 2018 ^79^ | 140 | Brazil | N/A | SF-36, Author developed measure for Scale of Basic Activities of Daily Living |
| McClellan 2010 ^80^ | 28,923 | US | N/A | Author developed |
| McKercher 2013 ^81^ | 49 | Australia | N/A | KDQOL-SF, EQ-5D |
| Mujais 2009 ^82^ | 1186 | US | N/A | KDQOL-SF |
| Nidhi 2019 ^83^ | 100 | India | N/A | ICQ |
| Oh 2019 ^84^ | 1622 | Korea | N/A | KDQOL-SF |
| Oh 2022 ^85^ | 1618 | Korea | N/A | KDQOL-SF |
| Oh 2017 ^86^ | 1844 | Korea | N/A | KDQOL-SF |
| Okoro 2022 ^87^ | 220 | Nigeria | N/A | 15D |
| Peng 2013 ^88^ | 57 | China | N/A | SF-36 |
| Pereira 2022 ^89^ | 33 | Brazil | N/A | SF-36 |
| Perlman 2005 ^90^ | 634 | US | N/A | SF-36 |
| Picard 2021 ^91^ | 50 | Canada | N/A | SF-36 |
| Piccoli 2020 ^92^ | 422 | Italy | N/A | WHOQOL-BREF |
| Rajan 2013 ^93^ | 22,273 | US | N/A | SF-36 |
| Seidel 2014 ^94^ | 173 | Germany | N/A | SF-36, LLFDI |
| Senanayake 2020 ^95^ | 1079 | Sir Lanka | N/A | KDQOL-SF |
| Sin 2022 ^96^ | 59 | Canada | N/A | NQOL |
| Sritarapipat 2012 ^97^ | 216 | Thailand | N/A | Modified Barthel Activities of Daily Living Index (BAI),Chula Activities of Daily Living Index (Chula ADL). Graphs - Physical function in BADL, IADL |
| Tabata 2022 ^98^ | 61 | Japan | N/A | EQ-5D-5L |
| Tajima 2010 ^99^ | 537 | Japan | N/A | EQ-5D(-3L) |
| Tannor 2019 ^100^ | 202 | Ghana | N/A | RAND-36 |
| Taptagaporn 2021 ^101^ | 258 | Thailand | N/A | KDQOL-SF |
| Tesfaye 2020 ^102^ | 101 | Australia | N/A | Katz ADL, Lawton and Brody IADL, KDQOL-36 |
| Thancharoen 2020 ^103^ | 379 | Thailand | N/A | EQ-5D-5L |
| Tsai 2017 ^104^ | 161 | Taiwan | N/A | WHOQOL-BREF |
| Tsai 2010 ^105^ | 568 | Taiwan | N/A | WHOQOL-BREF |
| van Haalen 2020 ^106^ | 5276 | France, Germany, Spain, Italy, the UK, the US, China | N/A | EQ-5D-3L, WPAI |
| van Haalen 2020 ^107^ | 1052 | China | N/A | WPAI-SHP |
| Wang 2021 ^108^ | 378 | China | N/A | Author developed IADL, BADL |
| Wang 2019 ^109^ | 1079 | China | N/A | KDQOL-36 |
| Wee 2016 ^110^ | 311 | Singapore | N/A | KDQOL-SF |
| Wilkinson 2021 ^111^ | 102 | UK | N/A | DASI |
| Wirkner 2022 ^112^ | 160 | Germany | N/A | KDQOL-SF |
| Xiong 2022 ^113^ | 558 | China | N/A | SF-36 |
| Yapa 2021 ^114^ | 886 | Sir Lanka | N/A | SF-36 |

15D: 15 Dimensions; BAI: Modified Barthel Activities of Daily Living Index; Chula ADL: Chula Activities of Daily Living Index; DASI: Duke Activity Status Index; EQ-5D: EuroQoL; FACT-An: Functional Assessment of Cancer Therapy – Anemia; FACIT-SP: Functional Assessment of Chronic Illness Therapy - Spiritual Well-Being; HAP: Human Activity Profile; ICECAP-A: ICEpop CAPability measure for Adults; ICQ: Illness cognition questionnaire; IPAQ: International Physical Activity Questionnaire (long form); ADL: Activities of Daily Living; KDQOL: Kidney Disease Quality of Life; KDQOL-36: Kidney Disease Quality of Life - 36-Item Questionnaire; KDQOL-SF: Kidney Disease Quality of Life - Short-Form Questionnaire; KDQ: Kidney Disease Questionnaire; K-ADL: Korean Activities of Daily Living; K-IADL: Korean Instrumental Activities of Daily Living; LLFDI: Late Life Function and Disability Instrument; IADL: Instrumental Activities of Daily Living; LASA: Linear Analog Scale Assessment; MOS: Medical Outcomes Study; NQOL: Nutrition Quality of Life; SF-6D: Short-Form 6 Dimension; SF-12: Short-Form 12-Item Questionnaire; SF-36: Short-Form 36-Item Questionnaire; SIP: The Sickness Impact Profile; WHOQOL-BREF: World Health Organisation Quality of Life Brief Version; WHOQOL-OLD FACETS: World Health Organisation Quality of Life for Older People covering six facets; WPAI: Work Productivity and Activity Impairment; WPAI-SHP: Work Productivity and Activity Impairment-Specific Health Problem; WSAS: Work and Social Adjustment Scale; FS developed by Lin: ; Martini bADLS: Martini developed Scale of Basic Activities of Daily Living; Wang et al. BADL: Wang et al. Basic Activities of Daily Living; Wang IADL: Wang developed Instrumental Activities of Daily Living.

**S3. Overview of all measurement properties**

| Measurement | Definition according to the COSMIN* taxonomy |
| --- | --- |
| Hypotheses testing | The extent of scores being consistent with the hypotheses ensuring there is measurement and construct validity. |
| Responsiveness | The ability of the measure to detect changes over time. |
| Internal consistency | The degree of correlation between different items in the measure. |
| Measurement error | Errors of scores, either systematic or random, that are not the result of true changes in the construct being measured. |
| Reliability | The reproducibility and consistency of a measure that is free from measurement error. |
| Construct validity | The extent to which the measure assesses the intended outcome. |
| Convergent validity | The extent to which two theoretically related measures have correlated results. |
| Discriminant validity | The extent to which two theoretically unrelated measures have dissimilar results. |
| Content validity (including face validity) | The extent to which the measure adequately reflects the construct being assessed. |
| Structural validity | The extent to which the instruments measurement scores reflect the dimension of the construct being measured. |
| Criterion validity | The extent to which the measure correlates to a gold standard. |
| Cross-cultural validity | The extent to which the performance of the outcomes of a translated or culturally adapted instrument adequately reflects the performance of the original version of the instrument. |

^*^ COnsensus-based Standards for the selection of health Measurement INstruments

**S4. Validation data of psychometric properties of measures that have been used to assess life participation in CKD.**

| Measure | Validity | Reliability |
| --- | --- | --- |
| EQ-5D-3L ^115^ | **Convergent validity:** The Spearman’s correlation coefficients between EQ-5D-3L index score and SF-36 Physical (0.28) and Mental (0.34) summary score indicated a significant association (<0.001), positive in direction and low in strength. Similarly, with the EQ-5D-5L VAS scores to the SF-36 Physical (0.21) and Mental (0.18) summary score.  **Known groups validity:** Both the EQ-5D-3L and SF-36 measures showed differentiation between participants who were depressed and not depressed and participants with and without psychological distress (p<0.001). The mean (SD) EQ-5D-5L Index and VAS score in depressed and not depressed participants was 0.41 (0.34) and 0.73 (0.22) respectively. Compared to SF-36 Physical summary scores were 33.64 (13.82) and 40.44 (15.24) and Mental summary scores were 37.68 (11.29) and 44.45 (12.28) in depressed and not depressed participants respectively. Similar results were shown in participants with and without psychological distress. | **Internal consistency:** Internal consistency reflected by Cronbach's α = 0.834. |
| FACT-An (Functional Assessment of Cancer Therapy – Anemia): (Population: (Non-dialysis = 145, dialysis = 59) initiated dialysis patients) ^116^* ^48^ | **Convergent validity:** The FACT-Fatigue (r=0.76) and Anemia subscale r=0.77 (p<0.001) strongly correlated with the SF-36 vitality subscale. However, weaker correlations were shown for FACT-fatigue (r=0.67) and Anemia (r=0.66) to SF-36 social functioning. Also, FACT-Fatigue (r=0.61) and Anemia (r=0.64) to SF-36 Role-physical. FACT-An scores were modestly correlated at baseline. At weeks 13/17 correlations between FACT-An Total, Fatigue and AnS scores were higher (r>0.30).  **Known groups validity:** All key domains of FACT-An and SF-36 had highly significant differences: the FACT-An scores split by the SF-36 Physical Functioning domain were: FACT-Anemia subscale score (mean 46.4, [SD 13.9]) vs 61.6 (12.2), the FACT Fatigue subscale 28.9 (10.8) vs. 39.7 (9.5), and the Total FACT-An score 118.3 (28.8) vs 145.0 (25.0), all p < 0.0001.  **Responsiveness:** The FACT-An total and subscale scores with the exception of social-wellbeing, were able to detect change in Hb level, which is seen as the scores improved as the Hb level stabilised (improvements at week 9 and relatively stable by week 13/17 in Hb level). Mean scores were higher for non-dialysis groups for FACT-An total score and all subscale scores. For example, functional wellbeing subscale: baseline: 0.22(p<0.05); week 9: 0.21(p<0.05); week 17: 0.29 (p<0.001). | **Internal consistency:** The FACT-An total scores and subscales demonstrated good to excellent Cronbach’s α coefficients (ranging from 0.79 to 0.95).  **Test-retest reliability:** All total score and subscales demonstrated acceptable re-test reliability (ranging from 0.72 to 0.88) with >0.6 intraclass correlation coefficient as the criteria^48^. |
| SF-6D (Short-Form 6 Dimension): (Population: 10% PD, 54%  HD, 36% conservative care) ^117^ | **Convergent validity:** Pearson’s coefficient was used to determine convergent validity with role (r=0.51, p<0.001) and control (r=0.53, p<0.001) domains of the ICECAP-O measure were strongly correlated with the pain domain of the SF-6D. All other domains of the ICECAP-O and SF-6D were weakly or moderately correlated (ranging from 0.02 to 0.49). | NA |
| SF-36 ^48^  (Population: (Non-dialysis = 145, dialysis = 59) | **Convergent validity:** SF-36 vitality domain strongly correlated with FACT-Fatigue (r=0.76) and Anemia subscale (r=0.77, p<0.001). Weaker correlations were shown for SF-36 Role physical to FACT-Fatigue (r=0.61) and Anemia (r=0.64) subscales. As well as SF-36 social functioning to FACT-Fatigue (r=0.67) and Anemia (r=0.66) subscales.  **Known groups validity:** All key domains of FACT-An and SF-36 had highly significant differences: the FACT-An scores split by the SF-36 Physical Functioning domain were: FACT-Anemia subscale score (mean 46.4, [SD 13.9]) vs 61.6 (12.2), the FACT Fatigue subscale 28.9 (10.8) vs. 39.7 (9.5), and the Total FACT-An score 118.3 (28.8) vs 145.0 (25.0), all p < 0.0001.  **Responsiveness:** The SF-36 domains and component score were able to detect changes in Hb level. For example, social functioning: baseline: 0.10 (p<0.05); week 9: 0.12 (p<0.001); week 17: 0.31 (p<0.001). However larger changes were seen in the dialysis group, only in the vitality domain saw an increase greater than three-points by week 9 (p<0.001) or 17 (p<0.01). | **Internal consistency:** The SF-36 domains and component summary score with the exception of the General Health Domain (0.69) demonstrated good to excellent Cronbach’s α coefficients (ranging from 0.76 to 0.93). For example, for Social Functioning (0.76), Role-Physical (0.93) and Physical Functioning (0.90).  **Test-retest reliability:** All domains and summary scores demonstrated re-test reliability (ranging from 0.64 to 0.83) with >0.6 intraclass correlation coefficient as an acceptable cut off ^48^. For example, for Social Functioning, Role-Physical and Physical Functioning with interclass correlation coefficient of 0.69, 0.69 and 0.83 respectively. |

*Abstract only available; EQ-5D: EuroQoL; FACT-An: Functional Assessment of Cancer Therapy – Anemia; SF-6D: Short-Form 6 Dimension; SF-36: Short-Form 36-Item Questionnaire; NA: Not available.

**Supplementary references**

1. Akizawa T, Gejyo F, Nishi S, et al. Positive Outcomes of High Hemoglobin Target in Patients With Chronic Kidney Disease Not on Dialysis: A Randomized Controlled Study. *Therapeutic Apheresis and Dialysis.* 2011;15(5):431-440.

2. Alexander M, Kewalramani R, Agodoa I, Globe D. Association of anemia correction with health related quality of life in patients not on dialysis. *Curr Med Res Opin.* 2007;23(12):2997-3008.

3. Arnold R, Pianta TJ, Pussell BA, et al. Randomized, Controlled Trial of the Effect of Dietary Potassium Restriction on Nerve Function in CKD. *Clinical Journal of The American Society of Nephrology: CJASN.* 2017;12(10):1569-1577.

4. Blakeman T, Blickem C, Kennedy A, et al. Effect of information and telephone-guided access to community support for people with chronic kidney disease: Randomised controlled trial. *PLoS ONE.* 2014;9(10) (no pagination).

5. Bohlke M, Barcellos FC, Santos IS, Mielke GI, de Marmann Vargas M, Hallal PC. Effects of a 16-week physical training on clinical outcomes in patients with hypertension and chronic kidney disease: NEPHROS post-trial follow-up. *Cadernos de Saude Publica.* 2022;38(5) (no pagination).

6. Campbell KL, Ash S, Bauer JD. The impact of nutrition intervention on quality of life in pre-dialysis chronic kidney disease patients. *Clinical Nutrition.* 2008;27(4):537-544.

7. Greenwood SA, Beckley-Hoelscher N, Asgari E, et al. The effect of intravenous iron supplementation on exercise capacity in iron-deficient but not anaemic patients with chronic kidney disease: study design and baseline data for a multicentre prospective double-blind randomised controlled trial. *BMC Nephrology.* 2022;23(1) (no pagination).

8. Hedayati SS, Gregg LP, Carmody T, et al. Effect of sertraline on depressive symptoms in patients with chronic kidney disease without dialysis dependence: The CAST randomized clinical trial. *JAMA - Journal of the American Medical Association.* 2017;318(19):1876-1890.

9. Hirakata H, Tsubakihara Y, Gejyo F, et al. Maintaining high hemoglobin levels improved the left ventricular mass index and quality of life scores in pre-dialysis Japanese chronic kidney disease patients. *Clin Exp Nephrol.* 2010;14(1):28-35.

10. Lewis EF, Pfeffer MA, Feng A, et al. Darbepoetin alfa impact on health status in diabetes patients with kidney disease: a randomized trial. *Clinical Journal of The American Society of Nephrology: CJASN.* 2011;6(4):845-855.

11. Li WY, Chiu FC, Zeng JK, et al. Mobile health app with social media to support self-management for patients with chronic kidney disease: Prospective randomized controlled study. *Journal of Medical Internet Research.* 2020;22(12) (no pagination).

12. Mager DR, Jackson ST, Hoffmann MR, Jindal K, Senior PA. Vitamin D<inf>3</inf> supplementation, bone health and quality of life in adults with diabetes and chronic kidney disease: Results of an open label randomized clinical trial. *Clinical Nutrition.* 2017;36(3):686-696.

13. Mustata S, Groeneveld S, Davidson W, Ford G, Kiland K, Manns B. Effects of exercise training on physical impairment, arterial stiffness and health-related quality of life in patients with chronic kidney disease: A pilot study. *International Urology and Nephrology.* 2011;43(4):1133-1141.

14. Provenzano R, Bhaduri S, Singh AK, Group PS. Extended epoetin alfa dosing as maintenance treatment for the anemia of chronic kidney disease: the PROMPT study. *Clin Nephrol.* 2005;64(2):113-123.

15. Roger SD, Jassal SV, Woodward MC, Soroka S, McMahon LP. A randomised single-blind study to improve health-related quality of life by treating anaemia of chronic kidney disease with Aranesp R (darbepoetin alfa) in older people: STIMULATE. *Int Urol Nephrol.* 2014;46(2):469-475.

16. Rossi AP, Burris DD, Lucas FL, Crocker GA, Wasserman JC. Effects of a renal rehabilitation exercise program in patients with CKD: a randomized, controlled trial. *Clinical Journal of The American Society of Nephrology: CJASN.* 2014;9(12):2052-2058.

17. Shin J, Hwang JH, Han M, et al. Phase angle as a marker for muscle health and quality of life in patients with chronic kidney disease. *Clinical Nutrition.* 2022;41(8):1651-1659.

18. Uchiyama K, Adachi K, Muraoka K, et al. Home-based aerobic exercise and resistance training for severe chronic kidney disease: a randomized controlled trial. *J Cachexia Sarcopenia Muscle.* 2021;12(6):1789-1802.

19. Van Craenenbroeck AH, Van Craenenbroeck EM, Van Ackeren K, et al. Effect of Moderate Aerobic Exercise Training on Endothelial Function and Arterial Stiffness in CKD Stages 3-4: A Randomized Controlled Trial. *American Journal of Kidney Diseases.* 2015;66(2):285-296.

20. Villar E, Lievre M, Kessler M, et al. Anemia normalization in patients with type 2 diabetes and chronic kidney disease: Results of the NEPHRODIAB2 randomized trial. *Journal of Diabetes and its Complications.* 2011;25(4):237-243.

21. Guo Y, Zhang M, Ye T, et al. Non-protein energy supplement for malnutrition treatment in patients with chronic kidney disease. *Asia Pacific journal of clinical nutrition.* 2022;31(3):504-511.

22. Kuo YT, Chiu KM, Tsang YM, Chiu CM, Chien MY. Influence of Chronic Kidney Disease on Physical Function and Quality of Life in Patients after Coronary Artery Bypass Grafting. *CardioRenal Medicine.* 2015;5(4):237-245.

23. Lefebvre P, Vekeman F, Sarokhan B, Enny C, Provenzano R, Cremieux PY. Relationship between hemoglobin level and quality of life in anemic patients with chronic kidney disease receiving epoetin alfa. *Current Medical Research and Opinion.* 2006;22(10):1929-1937.

24. Abeywickrama HM, Wimalasiri S, Koyama Y, et al. Quality of life and symptom burden among chronic kidney disease of uncertain etiology (CKDU) patients in Girandurukotte, Sri Lanka. *International Journal of Environmental Research and Public Health.* 2020;17(11):1-16.

25. Adame Perez SI, Senior PA, Field CJ, Jindal K, Mager DR. Frailty, Health-Related Quality of Life, Cognition, Depression, Vitamin D and Health-Care Utilization in an Ambulatory Adult Population With Type 1 or Type 2 Diabetes Mellitus and Chronic Kidney Disease: A Cross-Sectional Analysis. *Canadian Journal of Diabetes.* 2019;43(2):90-97.

26. Aggarwal HK, Jain D, Pawar S, Yadav RK. Health-related quality of life in different stages of chronic kidney disease. *Qjm.* 2016;109(11):711-716.

27. Alshelleh S, Alhouri A, Taifour A, et al. Prevelance of depression and anxiety with their effect on quality of life in chronic kidney disease patients. *Scientific reports.* 2022;12(1):17627.

28. Andrew A, Jose S, Sudha M, Venkateswaramurthy N, Sambath Kumar R, Shanmugasundaram R. Association of medication adherence with quality of life and treatment satisfaction among chronic kidney disease patients. *International Journal of Pharmaceutical Research.* 2020;13(1):1052-1059.

29. Arnold R, Pianta TJ, Issar T, et al. Peripheral neuropathy: An important contributor to physical limitation and morbidity in stages 3 and 4 chronic kidney disease. *Nephrology Dialysis Transplantation.* 2022;37(4):713-719.

30. Baddour NA, Robinson-Cohen C, Lipworth L, et al. The Surprise Question and Self-Rated Health Are Useful Screens for Frailty and Disability in Older Adults with Chronic Kidney Disease. *Journal of Palliative Medicine.* 2019;22(12):1522-1529.

31. Bagasha P, Namukwaya E, Leng M, et al. Comparison of the health-related quality of life of end stage kidney disease patients on hemodialysis and non-hemodialysis management in Uganda. *BMC Palliat Care.* 2021;20(1):52.

32. Belkin MD, Doerfler RM, Wagner LA, Zhan M, Fink JC. Associations of Performance-Based Functional Assessments and Adverse Outcomes in CKD. *Kidney360.* 2021;2(4):629-638.

33. Bonner A, Caltabiano M, Berlund L. Quality of life, fatigue, and activity in Australians with chronic kidney disease: a longitudinal study. *Nursing & health sciences.* 2013;15(3):360-367.

34. Bowling CB, Berkowitz TSZ, Smith B, et al. Unintended Consequences of COVID-19 Social Distancing Among Older Adults With Kidney Disease. *The journals of gerontology.* 2022;Series A, Biological sciences and medical sciences. 77(4):e133-e137.

35. Busa I, Ordonez-Mena JM, Yang Y, et al. Quality of life in older adults with chronic kidney disease and transient changes in renal function: Findings from the Oxford Renal cohort. *PLoS ONE.* 2022;17(10 October) (no pagination).

36. Chesnaye NC, Meuleman Y, de Rooij ENM, et al. Health-Related Quality-of-Life Trajectories over Time in Older Men and Women with Advanced Chronic Kidney Disease. *Clinical Journal of the American Society of Nephrology.* 2022;17(2):205-214.

37. Chin HJ, Ahn SY, Ryu J, et al. Renal function and decline in functional capacity in older adults. *Age & Ageing.* 2014;43(6):833-838.

38. Chin HJ, Song YR, Lee JJ, et al. Moderately decreased renal function negatively affects the health-related quality of life among the elderly Korean population: A population-based study. *Nephrology Dialysis Transplantation.* 2008;23(9):2810-2817.

39. Covic A, Jackson J, Hadfield A, Pike J, Siriopol D. Real-World Impact of Cardiovascular Disease and Anemia on Quality of Life and Productivity in Patients with Non-Dialysis-Dependent Chronic Kidney Disease. *Advances in Therapy.* 2017;34(7):1662-1672.

40. Davison SN, Jhangri GS, Feeny DH. Comparing the Health Utilities Index Mark 3 (HUI3) with the Short Form-36 preference-based SF-6D in chronic kidney disease. *Value in Health.* 2009;12(2):340-345.

41. de Pina Pereira RM, Aparecido Batista M, de Sousa Meira A, Pilotto de Oliveira M, Kusumota L. Qualidade de vida de idosos com doença renal crônica em tratamento conservador. *Revista Brasileira de Enfermagem.* 2017;70(4):887-895.

42. Delgado CE, Jaramillo MM, Orozco BE, et al. [Quality of life in patients with chronic kidney disease without dialysis or transplant: a random sample from two insurance companies. Medellin, Colombia, 2008]. *Nefrologia.* 2009;29(6):548-556.

43. Erez G, Selman L, Murtagh FE. Measuring health-related quality of life in patients with conservatively managed stage 5 chronic kidney disease: limitations of the Medical Outcomes Study Short Form 36: SF-36. *Quality of Life Research.* 2016;25(11):2799-2809.

44. Eriksson D, Goldsmith D, Teitsson S, Jackson J, Van Nooten F. Cross-sectional survey in CKD patients across Europe describing the association between quality of life and anaemia. *BMC Nephrology.* 2016;17(1) (no pagination).

45. Farag YM, Keithi-Reddy SR, Mittal BV, et al. Anemia, inflammation and health-related quality of life in chronic kidney disease patients. *Clin Nephrol.* 2011;75(6):524-533.

46. Feng L, Yap KB, Ng TP. Depressive symptoms in older adults with chronic kidney disease: mortality, quality of life outcomes, and correlates. *Am J Geriatr Psychiatry.* 2013;21(6):570-579.

47. Finkelstein FO, Story K, Firanek C, et al. Health-related quality of life and hemoglobin levels in chronic kidney disease patients. *Clinical Journal of the American Society of Nephrology.* 2009;4(1):33-38.

48. Finkelstein FO, van Nooten F, Wiklund I, Trundell D, Cella D. Measurement properties of the Short Form-36 (SF-36) and the Functional Assessment of Cancer Therapy - Anemia (FACT-An) in patients with anemia associated with chronic kidney disease. *Health and Quality of Life Outcomes.* 2018;16(1):111.

49. Fraser SD, Barker J, Roderick PJ, et al. Health-related quality of life, functional impairment and comorbidity in people with mild-to-moderate chronic kidney disease: a cross-sectional study. *BMJ open.* 2020;10(8):e040286.

50. Guedes M, Muenz D, Zee J, et al. Serum biomarkers of iron stores are associated with worse physical health-related quality of life (HRQoL) in non-dialysis dependent chronic kidney disease (NDD-CKD) patients with or without anemia. *Nephrology, dialysis, transplantation : official publication of the European Dialysis and Transplant Association European Renal Association.* 2021;24.

51. Gunawardena N, Palihawadana P, Senanayake S, Karunarathna R, Kumara P, Kularatna S. Health related quality of life in chronic kidney disease; A descriptive study in a rural Sri Lankan community affected by chronic kidney disease. *Health and Quality of Life Outcomes.* 2020;18(1) (no pagination).

52. Gyamlani G, Basu A, Geraci S, et al. Depression, screening and quality of life in chronic kidney disease. *American Journal of the Medical Sciences.* 2011;342(3):186-191.

53. Hakamaki M, Lankinen R, Hellman T, et al. Quality of Life Is Associated with Cardiac Biomarkers, Echocardiographic Indices, and Mortality in CKD Stage 4-5 Patients Not on Dialysis. *Blood Purification.* 2021;50(3):347-354.

54. Hamilton R, Hawley S. Quality of life outcomes related to anemia management of patients with chronic renal failure. *Clinical nurse specialist CNS.* 2006;20(3):139-143; quiz 144-145.

55. Hansen RA, Chin H, Blalock S, Joy MS. Predialysis chronic kidney disease: Evaluation of quality of life in clinic patients receiving comprehensive anemia care. *Research in Social and Administrative Pharmacy.* 2009;5(2):143-153.

56. Hao CM, Wittbrodt ET, Palaka E, Guzman N, Dunn A, Grandy S. Understanding patient perspectives and awareness of the impact and treatment of anemia with chronic kidney disease: A patient survey in China. *International Journal of Nephrology and Renovascular Disease.* 2021;14:53-64.

57. Ho YF, Hsu PT, Yang KL. The mediating effect of sleep quality and fatigue between depression and renal function in nondialysis chronic kidney disease: a cross-sectional study. *BMC Nephrol.* 2022;23(1):126.

58. Hoshino J, Muenz D, Zee J, et al. Associations of Hemoglobin Levels With Health-Related Quality of Life, Physical Activity, and Clinical Outcomes in Persons With Stage 3-5 Nondialysis CKD. *J Ren Nutr.* 2020;30(5):404-414.

59. Hussain S, Habib A, Najmi AK. Anemia prevalence and its impact on health-related quality of life in Indian diabetic kidney disease patients: Evidence from a cross-sectional study. *Journal of Evidence-Based Medicine.* 2019;12(4):243-252.

60. Jalal SM, Beth MRM, Bo Khamseen ZM. Impact of Hospitalization on the Quality of Life of Patients with Chronic Kidney Disease in Saudi Arabia. *Int J Environ Res Public Health.* 2022;19(15):07.

61. Jesky MD, Dutton M, Dasgupta I, et al. Health-Related Quality of Life Impacts Mortality but Not Progression to End-Stage Renal Disease in Pre-Dialysis Chronic Kidney Disease: A Prospective Observational Study. *PLoS ONE [Electronic Resource].* 2016;11(11):e0165675.

62. Jhamb M, Liang K, Yabes J, et al. Prevalence and correlates of fatigue in chronic kidney disease and end-stage renal disease: Are sleep disorders a key to understanding fatigue? *American Journal of Nephrology.* 2014;38(6):489-495.

63. Kaltsouda A, Skapinakis P, Damigos D, et al. Defensive coping and health-related quality of life in chronic kidney disease: a cross-sectional study. *BMC Nephrology.* 2011;12:28.

64. Kefale B, Alebachew M, Tadesse Y, Engidawork E. Quality of life and its predictors among patients with chronic kidney disease: A hospital-based cross sectional study. *PLoS ONE [Electronic Resource].* 2019;14(2):e0212184.

65. Kharshid AM, Sulaiman SAS, Saadh MJ. Health-related quality of life in chronic kidney disease patients: A cross-sectional study. *Systematic Reviews in Pharmacy.* 2020;11(7):188-192.

66. Kim HJ, Kim DW, Rhee H, et al. Rapid decline in kidney function is associated with rapid deterioration of health-related quality of life in chronic kidney disease. *Scientific reports.* 2023;13(1):1786.

67. Klang B, Bjorvell H, Berglund J, Sundstedt C, Clyne N. Predialysis patient education: effects on functioning and well-being in uraemic patients. *Journal of advanced nursing.* 1998;28(1):36-44.

68. Korevaar JC, Jansen MA, Merkus MP, Dekker FW, Boeschoten EW, Krediet RT. Quality of life in predialysis end-stage renal disease patients at the initiation of dialysis therapy. The NECOSAD Study Group. *Perit Dial Int.* 2000;20(1):69-75.

69. Kosmadakis GC, John SG, Clapp EL, et al. Benefits of regular walking exercise in advanced pre-dialysis chronic kidney disease. *Nephrology Dialysis Transplantation.* 2012;27(3):997-1004.

70. Krishnasamy R, Hawley CM, Stanton T, et al. Association between left ventricular global longitudinal strain, health-related quality of life and functional capacity in chronic kidney disease patients with preserved ejection fraction. *Nephrology.* 2016;21(2):108-115.

71. Kularatna S, Senanayake S, Gunawardena N, Graves N. Comparison of the EQ-5D 3L and the SF-6D (SF-36) contemporaneous utility scores in patients with chronic kidney disease in Sri Lanka: A Cross-sectional survey. *BMJ Open.* 2019;9(2) (no pagination).

72. Kusek JW, Greene P, Wang SR, et al. Cross-sectional study of health-related quality of life in African Americans with chronic renal insufficiency: The African American Study of Kidney Disease and Hypertension Trial. *American Journal of Kidney Diseases.* 2002;39(3):513-524.

73. Lee J, Abdel-Kader K, Yabes JG, Cai M, Chang HH, Jhamb M. Association of Self-Rated Health With Functional Limitations in Patients With CKD. *Kidney Medicine.* 2021;3(5):745-752.e741.

74. Lee YJ, Kim MS, Cho S, Kim SR. Association of depression and anxiety with reduced quality of life in patients with predialysis chronic kidney disease. *International Journal of Clinical Practice.* 2013;67(4):363-368.

75. Lemos CF, Rodrigues MP, Veiga JR. Family income is associated with quality of life in patients with chronic kidney disease in the pre-dialysis phase: a cross sectional study. *Health & Quality of Life Outcomes.* 2015;13:202.

76. Lidgard B, Zelnick LR, O'Brien K D, Bansal N. Patient-Reported Symptoms and Subsequent Risk of Myocardial Infarction in Chronic Kidney Disease. *Clinical Journal of the American Society of Nephrology.* 2022;17(4):487-495.

77. Lin SF, Fan YC, Kuo TT, Pan WH, Bai CH. Quality of life and cognitive assessment in healthy older Asian people with early and moderate chronic kidney disease: The NAHSIT 2013-2016 and validation study. *PLoS ONE.* 2022;17(3 March) (no pagination).

78. Mansur HN, Colugnati FA, Grincenkov FR, Bastos MG. Frailty and quality of life: a cross-sectional study of Brazilian patients with pre-dialysis chronic kidney disease. *Health & Quality of Life Outcomes.* 2014;12:27.

79. Martini A, Ammirati A, Garcia C, et al. Evaluation of quality of life, physical, and mental aspects in longevous patients with chronic kidney disease. *International Urology and Nephrology.* 2018;50(4):725-731.

80. McClellan WM, Abramson J, Newsome B, et al. Physical and psychological burden of chronic kidney disease among older adults. *American Journal of Nephrology.* 2010;31(4):309-317.

81. McKercher CM, Venn AJ, Blizzard L, et al. Psychosocial factors in adults with chronic kidney disease: Characteristics of pilot participants in the Tasmanian Chronic Kidney Disease study. *BMC Nephrology.* 2013;14(1) (no pagination).

82. Mujais SK, Story K, Brouillette J, et al. Health-related quality of Life in CKD patients: Correlates and evolution over time. *Clinical Journal of the American Society of Nephrology.* 2009;4(8):1293-1301.

83. Nidhi R, Prudhivi R, Lal L, et al. A prospectivestudy on assessment of acceptance and functional capacity of chronic kidney disease patients in tertiary care hospital. *Journal of Pharmaceutical Sciences and Research.* 2019;11(9):3281-3288.

84. Oh TR, Choi HS, Kim CS, et al. Association between health related quality of life and progression of chronic kidney disease. *Scientific Reports.* 2019;9(1):19595.

85. Oh TR, Choi HS, Suh SH, et al. The Association between Health-Enhancing Physical Activity and Quality of Life in Patients with Chronic Kidney Disease: Propensity Score Matching Analysis. *International Journal of Environmental Research and Public Health.* 2022;19(3) (no pagination).

86. Oh TR, Kim CS, Bae EH, et al. Association between vitamin D deficiency and health-related quality of life in patients with chronic kidney disease from the KNOW-CKD study. *PLoS ONE [Electronic Resource].* 2017;12(4):e0174282.

87. Okoro RN, Adibe MO, Okonta MJ, Ummate I, Ohieku JD, Yakubu S. Assessment of health-related quality of life and its determinants in the pre-dialysis patients with chronic kidney disease. *Int Urol Nephrol.* 2022;54(1):165-172.

88. Peng T, Hu Z, Guo L, Xia Q, Li D, Yang X. Relationship between psychiatric disorders and quality of life in nondialysis patients with chronic kidney disease. *American Journal of the Medical Sciences.* 2013;345(3):218-221.

89. Pereira RA, Alvarenga MDS, de Andrade LS, et al. Effect of a nutritional behavioral intervention on intuitive eating in overweight women with chronic kidney disease. *Journal of renal nutrition : the official journal of the Council on Renal Nutrition of the National Kidney Foundation.* 2022;02.

90. Perlman RL, Finkelstein FO, Liu L, et al. Quality of life in Chronic Kidney Disease (CKD): A cross-sectional analysis in the Renal Research Institute-CKD study. *American Journal of Kidney Diseases.* 2005;45(4):658-666.

91. Picard K, Senior PA, Adame Perez S, Jindal K, Richard C, Mager DR. Low Mediterranean Diet scores are associated with reduced kidney function and health related quality of life but not other markers of cardiovascular risk in adults with diabetes and chronic kidney disease. *Nutrition, Metabolism and Cardiovascular Diseases.* 2021;31(5):1445-1453.

92. Piccoli GB, B.R DII, Chatrenet A, et al. Dietary satisfaction and quality of life in chronic kidney disease patients on low-protein diets: A multicentre study with long-term outcome data (TOrino-Pisa study). *Nephrology Dialysis Transplantation.* 2020;35(5):790-802.

93. Rajan M, Lai KC, Tseng CL, et al. Estimating utilities for chronic kidney disease, using SF-36 and SF-12-based measures: challenges in a population of veterans with diabetes. *Quality of life research : an international journal of quality of life aspects of treatment, care and rehabilitation.* 2013;22(1):53-64.

94. Seidel UK, Gronewold J, Volsek M, et al. Physical, cognitive and emotional factors contributing to quality of life, functional health and participation in community dwelling in chronic kidney disease. *PLoS ONE.* 2014;9(3) (no pagination).

95. Senanayake S, Gunawardena N, Palihawadana P, et al. Health related quality of life in chronic kidney disease; a descriptive study in a rural Sri Lankan community affected by chronic kidney disease. *Health & Quality of Life Outcomes.* 2020;18(1):106.

96. Sin D, Harasemiw O, Curtis S, et al. Dietary Patterns and Perceptions in Older Adults With Chronic Kidney Disease in the Canadian Frailty Observation and Interventions Trial (CanFIT): A Mixed-Methods Study. *Canadian Journal of Kidney Health and Disease.* 2022;9(no pagination).

97. Sritarapipat P, Pothiban L, Panuthai S, Lumlertgul D, Nanasilp P. Causal Model of Elderly Thais' Self-Management Behaviors of Pre-dialysis Chronic Kidney Disease. *Pacific Rim International Journal of Nursing Research.* 2012;16(4):277-293.

98. Tabata A, Yabe H, Katogi T, et al. Factors affecting health-related quality of life in older patients with chronic kidney disease: a single-center cross-sectional study. *International Urology and Nephrology.* 2022;54(10):2637-2643.

99. Tajima R, Kondo M, Kai H, et al. Measurement of health-related quality of life in patients with chronic kidney disease in Japan with EuroQol (EQ-5D). *Clin Exp Nephrol.* 2010;14(4):340-348.

100. Tannor EK, Norman BR, Adusei KK, Sarfo FS, Davids MR, Bedu-Addo G. Quality of life among patients with moderate to advanced chronic kidney disease in Ghana - A single centre study. *BMC Nephrology.* 2019;20(1) (no pagination).

101. Taptagaporn S, Mongkolsomlit S, Rakkapao N, Kaewdok T, Wattanasoei S. Quality of life among patients suffering from chronic kidney disease in chronic kidney disease clinic of thailand. *Open Public Health Journal.* 2021;14(1):417-424.

102. Tesfaye WH, McKercher C, Peterson GM, et al. Medication adherence, burden and health-related quality of life in adults with predialysis chronic kidney disease: A prospective cohort study. *International Journal of Environmental Research and Public Health.* 2020;17(1) (no pagination).

103. Thancharoen O, Waleekhachonloet O, Limwattananon C, Anutrakulchai S. Cognitive impairment, quality of life, and healthcare utilization in patients with chronic kidney disease stages 3-5. *Nephrology.* 2020;05.

104. Tsai YC, Chen HM, Hsiao SM, et al. Association of physical activity with cardiovascular and renal outcomes and quality of life in chronic kidney disease. *PLoS ONE [Electronic Resource].* 2017;12(8):e0183642.

105. Tsai YC, Hung CC, Hwang SJ, et al. Quality of life predicts risks of end-stage renal disease and mortality in patients with chronic kidney disease. *Nephrology Dialysis Transplantation.* 2010;25(5):1621-1626.

106. van Haalen H, Jackson J, Spinowitz B, Milligan G, Moon R. Impact of chronic kidney disease and anemia on health-related quality of life and work productivity: analysis of multinational real-world data. *BMC Nephrology.* 2020;21(1):1-15.

107. van Haalen H, Sloand J, Moon R, et al. Drug treatment patterns and work productivity in chronic kidney disease patients with anemia in China: Cross sectional analysis of real-world data. *Kidney Research and Clinical Practice.* 2020;39(3):318-333.

108. Wang M, Li L, Wang K. Risk factors for reduced quality of lifein ckd patients over 65 years old. *Acta Medica Mediterranea.* 2021;37(6):3537-3540.

109. Wang WL, Liang S, Zhu FL, et al. The prevalence of depression and the association between depression and kidney function and health-related quality of life in elderly patients with chronic kidney disease: A multicenter cross-sectional study. *Clinical Interventions in Aging.* 2019;14:905-913.

110. Wee HL, Seng BJ, Lee JJ, et al. Association of anemia and mineral and bone disorder with health-related quality of life in Asian pre-dialysis patients. *Health & Quality of Life Outcomes.* 2016;14:94.

111. Wilkinson TJ, Gore EF, Baker LA, Watson EL, Smith AC. Muscle power and physical dysfunction: A model for tailoring rehabilitation in chronic kidney disease. *Nephrology.* 2021;26(10):790-797.

112. Wirkner J, Scheuch M, Dabers T, et al. Comorbid Depression and Diabetes Are Associated with Impaired Health-Related Quality of Life in Chronic Kidney Disease Patients. *Journal of Clinical Medicine.* 2022;11(16) (no pagination).

113. Xiong J, Peng H, Yu Z, et al. Daily Walking Dose and Health-related Quality of Life in Patients With Chronic Kidney Disease. *Journal of renal nutrition : the official journal of the Council on Renal Nutrition of the National Kidney Foundation.* 2022;32(6):710-717.

114. Yapa HE, Purtell L, Chambers S, Bonner A. Alterations in symptoms and health-related quality of life as kidney function deteriorates: A cross-sectional study. *J Clin Nurs.* 2021;30(11-12):1787-1796.

115. Senanayake S, Mahesh PKB, Gunawardena N, Graves N, Kularatna S. Validity and internal consistency of EQ-5D-3L quality of life tool among pre-dialysis patients with chronic kidney disease in Sri Lanka, a lower middle-income country. *PLOS ONE.* 2019;14(6):e0211604.

116. van Nooten FE, Wiklund I, Trundell D, Cella D. PSYCHOMETRIC EVALUATION OF THE FUNCTIONAL ASSESSMENT OF CANCER THERAPY&#x2013;ANEMIA (FACT-AN) IN DIALYSIS AND NON-DIALYSIS PATIENTS WITH ANEMIA ASSOCIATED WITH CHRONIC KIDNEY DISEASE. *Value in Health.* 2016;19(3):A91.

117. Shah K, Murtagh F, McGeechan K, et al. Health-related quality of life and well-being in people over 75 years of age with end-stage kidney disease managed with dialysis or comprehensive conservative care: A cross-sectional study in the UK and Australia. *BMJ Open.* 2019.
